# Vigorous Intervals and Hypoglycemia in Type 1 Diabetes: A Randomized Cross Over Trial

**DOI:** 10.1038/s41598-018-34342-6

**Published:** 2018-10-26

**Authors:** Meaghan Rempel, Jane E. Yardley, Andrea MacIntosh, Jacqueline L. Hay, Danielle Bouchard, Stephen Cornish, Seth D. Marks, Yan Hai, Joseph W. Gordon, Jonathan McGavock

**Affiliations:** 1 Department of Pediatrics and Child Health, Rady Faculty of Health Sciences, University of Manitoba, Winnipeg, Manitoba Canada; 2 Children’s Hospital Research Institute of Manitoba, Winnipeg, Manitoba Canada; 3 University of Alberta, Augustana Faculty, Camrose, Alberta, Canada; 4 University of Alberta, Faculty of Kinesiology, Sport and Recreation, Alberta, Canada; 5 Faculty of Kinesiology, University of New Brunswick, New Brunswick, Canada; 6 Diabetes Education and Resource for Children and Adolescents, Children’s Hospital Health Sciences Centre, Winnipeg, Manitoba Canada; 7 Faculty of Kinesiology and Recreation Management, University of Manitoba, Winnipeg, Manitoba Canada; 8 College of Nursing, University of Manitoba, Winnipeg, Manitoba Canada; 9 Diabetes Research Envisioned and Accomplished in Manitoba (DREAM), Winnipeg, Manitoba Canada; 10 Department of Human Anatomy and Cell Science, Rady Faculty of Health Sciences, University of Manitoba, Winnipeg, Manitoba Canada

## Abstract

Adding vigorous-intensity intervals (VII) to moderate-intensity exercise prevents immediate declines in blood glucose in type 1 diabetes (T1D) however the intensity required to minimize post-exercise hypoglycemia is unknown. To examine this question, ten sedentary T1D individuals completed four treadmill exercise sessions: a control session of 45 minutes of walking at 45–55% of heart rate reserve (HRR) and three sessions consisting of 60 seconds (VII) at 70%, 80%, or 90% of HRR every 4 minutes during exercise at 45–55% of HRR. We used continuous glucose monitoring (CGM) to measure time ≤3.9 mmol/L, glucose variability, hypoglycemia frequency and area under the curve (AUC) for hypoglycemia and hyperglycemia for 12 hours post-exercise. We also examined growth hormone and cortisol responses during and following exercise. In the 12 hours post-exercise, the percentage of time ≤3.9 mmol/L, glucose variability, and AUC for hypoglycemia and hyperglycemia were similar across conditions. The frequency of hypoglycemic events was highest after the 90% intervals compared to the control arm (12 vs 3 events, p = 0.03). There was a trend towards elevated growth hormone with increasing exercise intensity but cortisol levels were similar across conditions. Adding VII to moderate intensity exercise may increase hypoglycemia risk at higher intensities.

## Introduction

Fear of exercise-induced hypoglycemia is a primary deterrent for engaging in an active lifestyle for individuals with type 1 diabetes (T1D)^1–3^. Several conventional clinical recommendations for preventing exercise-induced hypoglycemia focus on decreasing insulin administration prior to exercise and increasing exogenous carbohydrate intake prior to, during and/or following exercise^4–6^. However, it is frequently difficult to predict when there will be time available for exercise, leaving the latter as the sole option for many individuals. This approach may be counter-productive for those living with T1D who are using exercise as a strategy for weight maintenance and/or weight loss, and can also contribute to unwanted variability in blood glucose.

Adding vigorous intensity intervals to moderate intensity endurance exercise is a potential alternative to consuming exogenous glucose for preventing hypoglycemia for individuals with T1D. Vigorous intensity intervals stimulate a counter-regulatory hormonal response that can attenuate declines in blood glucose during^7–11^ and following exercise^12^ in individuals with T1D. This strategy is, therefore, a promising, calorie-neutral, approach to preventing exercise-related hypoglycemia, however its effectiveness remains unclear for several reasons. Most trials of vigorous intensity intervals for individuals with T1D were conducted in highly controlled settings, in the mid-morning, with participants beginning exercise within a narrow blood glucose range after standardized meals^7,8,10,11,13^. Of those performing afternoon exercise, few quantified post-exercise glucose levels during nocturnal hours^12,14^. Previous experiments were also performed mostly on stationary cycle ergometers, which is a less common form of activity for achieving daily activity targets. As people with T1D perform exercise more often in the afternoon^2^, mostly in the form of walking^2^ or running^15^, and with highly variable pre-exercise blood glucose levels^15^, the practical application of adding vigorous intensity intervals to moderate intensity exercise for preventing hypoglycemia remains unclear. Whether the counter-regulatory response to high intensity activity is able to provide a protective effect in the hours post-exercise, when glycogen stores are being replenished, is still a matter of debate.

Measures of glucose variability rely on frequent, systematic glucose monitoring^16,17^ and provide a sensitive, quantifiable measure of glycaemic variance over a given time period. For example, while two individuals may display similar HbA_1c_ levels, they may differ significantly in the frequency or magnitude of glucose dispersion from fasting levels, particularly in the post-prandial period^17,18^. Most importantly, glucose variability is a robust predictor of hypoglycemia in T1D individuals^19–21^. The impact of vigorous intensity intervals on post-exercise glucose variability has never been explored in individuals with T1D.

The purpose of this study was to address these gaps in the literature and determine the impact of vigorous intensity interval exercise on the risk of nocturnal hypoglycemia and glucose variability in individuals with T1D. Previous acute studies of intermittent vigorous intensity activity seem to have had the greatest success in preventing declines in blood glucose during exercise when the exercise intensity was either maximal^7–10,13^, or near maximal^12,22^. We therefore hypothesized that adding one-minute bouts of vigorous intensity exercise, above 80% of maximal aerobic power, to a moderate intensity endurance exercise session would significantly reduce the risk of nocturnal hypoglycemia and post-exercise glucose variability compared to a session of moderate intensity endurance exercise alone.

## Methods

### Design

The study hypothesis was tested using a randomized cross-over trial design, in which all participants performed all three experimental arms and a control condition. Conditions were performed in random order, with at least 48 hours between sessions. Intervention arms consisted of 40 minutes of moderate intensity endurance exercise (walking) interspersed with six one-minute intervals of vigorous intensity running at either 70, 80 or 90% of heart rate reserve (HRR) (every four minutes). The control condition consisted of 45 minutes of continuous moderate intensity endurance exercise (walking) at 45–55% of HRR. The primary outcome was determined for 12 hours following each acute exercise session using continuous glucose monitoring (CGM) data. To reflect a real-world setting, we asked participants to consume a diet of their choice prior to the initial exercise session (repeating that same diet for each session), provided sessions in the afternoon (17h00), and delivered exercise in the form of walking/running, rather than cycling. All participants provided written informed consent and the study was approved by the Biomedical Research Board at the University of Manitoba, Bannatyne Campus, in accordance with the Declaration of Helsinki. This trial is registered with the ClinicalTrials.gov Trial Registry Platform (NCT 03583268; posted 11/07/2018) and the study protocol is provided in the supplement.

### Participants

Between October 2013 and December 2015 we recruited 10 individuals (4 females) living with T1D by targeting local medical clinics and pharmacies in Winnipeg, Manitoba, Canada. To enhance external validity and limit variability in the outcome measures, we restricted recruitment to participants that had lived with T1D for at least 2 years who, according to self-report, were not currently meeting recommended daily physical activity levels [150 minutes of moderate to vigorous physical activity (MVPA) weekly for those aged 18+^23^, 60 minutes per day for those aged 15–17 years^23^]. We also restricted the age range for recruitment to those between 15 and 35 years of age, as younger individuals are known to have higher catecholamine responses to exercise^24^ and would best demonstrate the impact of VII on blood glucose. Their most recent HbA_1c_ was required to be ≤9.9% (85 mmol/mol) to ensure that they were performing appropriate self-management. Participants were excluded based on the following additional criteria: (1) frequent and unpredictable hypoglycemia, defined as either a severe low requiring assistance within the last three months or hypoglycemia unawareness; (2) unable to exercise due to injury or other restriction; (3) a change in insulin management regime in the past three months; (4) conditions that would make vigorous exercise unsafe; (5) taking atypical antipsychotics or corticosteroids that would interfere with glucose metabolism; (6) taking beta blockers; (7) being pregnant, breastfeeding, or planning on becoming pregnant; and (8) shift work requiring being awake at night.

### Baseline Screening

Anthropometric measures, medical history and HbA_1c_ were assessed during baseline screening. Peak oxygen uptake (VO_2peak_) was determined using a graded maximal exercise test to exhaustion on a treadmill with expired gas collection (ParvoMedics TrueOne Metabolic System – OUSW 4.2cx; ParvoMedics Salt Lake, UT). Upon completion of the maximal exercise test, a blinded continuous glucose monitor (CGM: iPro2^TM^, Medtronic Minimed) was inserted by a nurse into the abdomen or upper gluteal area to measure interstitial glucose levels over a six day period. Each participant recorded capillary blood glucose at least four times per day (ideally before each meal and before bedtime for maximum glucose stability), meal and total carbohydrate estimation, and daily insulin dose for all days of CGM wear. Participants self-selected their daily diet, but were asked to replicate their food intake as closely as possible on experimental days.

### Experimental Protocol

All participants arrived at the exercise laboratory at 16h00, having worn a CGM for at least 48 hours prior to testing. The randomization sequence for the conditions, developed by a statistician not involved in data collection, was allocated by a research assistant prior to the first scheduled experimental session. The research assistant, investigator team and participant were blinded to the allocation prior to the first visit as it was provided electronically. The research assistant involved in assigning the participants to the condition sequence was also involved in data collection.

Participants using multiple daily insulin injections were asked to decrease their long-acting insulin dose by 10% the night before and/or the morning of the exercise session and adjust accordingly with fast acting insulin correction doses throughout the day to avoid excessive hyperglycemia. Those using insulin pumps were asked to decrease their basal rate by 50% two hours before exercise, and maintain reduced basal rates until the end of exercise. Upon arrival for all exercise sessions, participants consumed a Glucerna Bar (Glucerna^TM^ Chocolate Graham Snack Bars, 150 calories, 25 g of carbohydrate) as other studies have done^12,25,26^. Glucerna^TM^ Nutritional Drinks (200 calories, 26 g of carbohydrate), were also provided as a bedtime snack for each exercise session, as others have done previously^12^. These methods are also consistent with clinical guideline recommendations from Diabetes Canada^27^. A blood glucose level between 5.7 and 13.9 mmol/l was required to start an experimental condition. Capillary glucose was measured 30 minutes before exercise and immediately before the start of exercise. If capillary glucose levels were below 5.7 mmol/l prior to exercise, participants were provided with glucose (Dex4^TM^, AMG Medical, Montreal, QC, Canada) and only started exercising once capillary glucose was >5.7 mmol/L. An IV catheter was inserted into the anticubital space or dorsal hand vein prior to exercise. Blood was sampled from the IV at baseline (0 mins), end of warm up (10 mins), end of intervals (35 mins), end of exercise (45 mins), mid-recovery (75 mins), and end-recovery (105 mins).

### Control Condition

The control condition consisted of moderate intensity exercise at 45–55% of HRR in the form of continuous walking for 45 minutes on a treadmill.

### Intervention Conditions

The experimental conditions consisted of vigorous intensity intervals lasting 60 seconds, with four minutes of recovery (at 45–55% of HRR), repeated six times. These intervals were preceded by a 10-minute warm-up, and followed by a 9-minute cool-down, also performed at 45–55% of HRR (45 minutes total). Participants returned on three separate occasions, with the vigorous intensity intervals set at 70%, 80%, or 90% of HRR for the respective sessions. The goal for each session was to select a workload that would elicit a heart rate and perceived effort that would fall into the target range during the last half of the intervals. The vigorous intensities selected corresponded to intensities that approached and exceeded ventilatory threshold, but did not exceed workloads associated with 100% of peak oxygen uptake. These intensities are considered vigorous according to the American College of Sports Medicine guidelines^28^.

### Outcome Measures

The primary outcome measure was time spent in hypoglycemia (≤3.9 mmol/L), during the 12 hours following exercise. This particular window of time was chosen as previous exercise studies have found differences among exercise conditions most notable during this window^12,26,29^, and less so during the full 24-hours post-exercise. The secondary outcome measures were measures of glucose variability^17,18^: mean absolute glucose change (MAG) and continuous overall net glycemic action (CONGA)^17,18^ at 1, 2, and 4 hours post-exercise. Mean absolute glucose change (MAG) is measured in mmol/L/h and is an assessment of glucose variability among exercise days. It is calculated by summing the absolute increments and decrements (even if they are in the physiological range) of glucose from peaks to nadirs within each hour, and has been validated as an index of glucose variability for closely spaced blood glucose measurements^30^. Continuous overall net glycemic action (CONGA_*n*_) is a measure of glucose variability that assesses changes in blood glucose values within the same day and is measured in mmol/L. It is determined by calculating the 24-hour mean of differences between CGM glucose data points and their corresponding point *n* hours earlier, with *n* being anywhere from 1 to 8 hours^31^.

Primary and secondary outcome measures were quantified using CGM [Enlite sensors, iPro2^TM^, Medtronic, Northridge, CA], which provides reliable interstitial glucose readings during and after both high intensity and continuous exercise in individuals with T1D^32^. CGM sensors were inserted subcutaneously during the baseline screening visit, which occurred 48 hours prior to the first exercise session. Capillary glucose readings during the exercise sessions in the laboratory were obtained from OneTouch UltraSmart handheld glucose meters (LifeScan; Johnson & Johnson, Milpitas, CA) and coded strips (same code throughout the study). Participants were asked to test capillary glucose with their own glucometer four times daily for calibration. CGM blinding ensured that participants could not alter dietary patterns or activity behavior based on real-time glucose monitoring. Twenty-four hours after the last session, CGM units were removed and data were uploaded to a server supported by Medtronic (Minimed Solutions v.3.0c; Medtronic, Northridge, CA). Raw data points were extracted following each session and uploaded into R (R 3.3.3 binary for Mac OS X 10.9) to calculate outcome measures.

Exploratory outcome measures included the frequency of hypoglycemic events, growth hormone and cortisol levels during and immediately following exercise, as well as the area under the curve (AUC) for hypoglycemia (≤3.9 mmol/L) and hyperglycemia (≥11.0 mmol/L) as measured in previous studies^25,26^. Plasma glucose was measured to confirm CGM accuracy, and plasma insulin was determined to eliminate this hormone as a potential confounder. Hypoglycemic events were defined as any interstitial glucose reading ≤3.9 mmol/L, with a minimum duration of 15 minutes, where individual events had to be separated by at least 30 minutes. Events were identified using CGM data based on the reporting recommendations of Schnell *et al*.^33^. Blood samples were collected in EDTA and serum tubes and centrifuged upon collection. Serum samples to be analysed for growth hormone levels were then stored at −80 °C for batch analysis, while analyses of plasma samples for cortisol, insulin and glucose were performed immediately. Glucose was measured using a two-point end enzymatic assay with hexokinase. Serum growth hormone was measured using a high sensitivity human growth hormone ELISA kit (Anogen, Mississauga, Ontario) with a sensitivity of 0.5 ng/mL. Plasma cortisol concentrations were also determined by ELISA (Cayman Chemical, Ann Arbor, Michigan). Assay sensitivity was 70 pg/mL. Hemoglobin A_1c_ was assessed using a Roche Cobas Integra 800 CTS analyzer (Roche Diagnostics, Indianapolis, IN). An Immulite (Siemens) solid-phase, two-site chemiluminescent immunometric assay was used to measure plasma insulin concentrations.

### Statistical Analyses

Non-normally distributed data are presented as median and percentiles (Q1–Q3). Normally distributed data are presented as mean and standard deviation. A Friedman test (non-parametric) and repeated measures ANOVA (parametric) were used for primary and secondary outcome measures, while all other analyses used a Friedman test only. A sample of 10 participants was sufficient to detect a 50% difference in the time spent in hypoglycemia between the sessions that included high intensity intervals and the 45–55% HRR session, assuming a β = 0.2 and α = 0.017 (adjusted for three comparisons). The projected 50% reduction in the time spent in hypoglycemia was derived from a previous acute study using a similar protocol to examine nocturnal hypoglycemia^12^ and is considered clinically relevant. All statistical analyses were conducted using Statistical Package for Social Science (SPSS) software version 24 (IBM, Chicago, ILL).

## Results

We screened 54 individuals with T1D for participation in the study between October 2013 and December 2015; 5 declined participation, 19 failed to follow up after initial contact, 20 did not meet one or more inclusion criteria. Ten participants (6 males, 4 females) completed all study protocols. Participant characteristics are listed in Table 1. Participants were on average 30 years old (ranging from 20 to 34) with a median duration of diabetes of 9.5 years. The majority of participants were male (60%) with a median VO_2_peak of 40.5 ml/kg/min and a median HbA_1c_ of 8.0%. All but one participant was on multiple daily injections of insulin.

**Table 1:** Participant characteristics.

Variable	Median (Q1–Q3) or N (%)
Age (years)	30 (25–32)
Sex (male)	6 (60)
Height (cm)	178.2 (167.9–183.5)
Weight (kg)	88.4 (76.6–95.1)
BMI (kg/m^2^)	27.6 (23.2–31.0)
Resting HR (bpm)	87 (81–96)
VO_2_peak (ml/kg/min)	37.6 (30.5–41.0)
Diabetes Duration (years)	9.5 (5.3–15.8)
Insulin Regimen MDI (%)	9 (90)
Total Daily Insulin Dose (IU)	52.3 (42.9–57.6)
HbA_1c_ (%)	8.0 (7.0–8.6)
HbA_1c_ (mmol/mol)	63.9 (53.0–70.5)

Results are presented as median and quartiles (Q1–Q3) or N (%). HR (heart rate), bpm (beats per minute), MDI (multiple daily injections), IU (international units). Insulin regimen for other participants was continuous subcutaneous insulin infusion.

### Exercise Sessions

All participants reached the prescribed exercise intensities by the end of each exercise session: (1) control condition: 58.1% of HRR; experimental condition 1 (70% of HRR): 73.7% of HRR, experimental condition 2 (80% of HRR): 84.4% of HRR, and experimental condition 3 (90% of HRR): 88.9% of HRR (Table 2). There were no significant differences among conditions in baseline blood glucose values (*p* = 0.22), baseline insulin concentrations (*p* = 0.07) or amount of carbohydrate (CHO) supplementation required before or during exercise, (*p* = 0.56) (Table 3). Testing day total carbohydrate intake (p = 0.599), insulin dosage (p = 0.168) and insulin to carbohydrate ratio (p = 0.887) were also not different across experimental and control conditions. There were no significant differences in median blood glucose levels during exercise in all conditions (*p* = 0.39). Finally, there was no significant change in blood glucose levels during exercise (*p* = 0.66) or change in blood glucose levels one hour post-exercise (*p* = 0.46) (Table 4).

**Table 2:** Heart rate reserve and rate of perceived exertion during exercise for each study arm.

Study Arm (%HRR)	Median %HRR	Median RPE
Control (45–55%)	58.1 (53.6–62.3)	12 (11–12)
Condition 1 (70%)	73.7 (70.2–79.0)	13 (13–13)
Condition 2 (80%)	84.4 (78.9–87.0)	14 (13–14)
Condition 3 (90%)	88.9 (83.0–92.2)	16 (15–16)

Data are presented as median and quartiles (Q1–Q3). HRR (heart rate reserve), RPE (rate of perceived exertion). Median RPE and median HRR for the interval sessions (70, 80, and 90%) are for the high intensity intervals only.

**Table 3:** Glucose values, carbohydrate intake and insulin adjustments across the study arms.

Study Arm (% HRR)	Baseline Glucose (mmol/L)	Insulin Prior to Exercise* (U/kg)	CHO Prior to Exercise (g)		CHO During Exercise (g)	Number of Participants Requiring CHO During Exercise
45–55	5.8 (4.9–7.0)	0.27 (0.21–0.43)		0 (0–66)	0 (0–44)	3
70	8.7 (6.2–9.4)	0.22 (0.16–0.26)		0 (0–24)	0 (0–12)	2
80	7.3 (6.2–8.6)	0.24 (0.19–0.33)		0 (0–8)	0 (0–44)	1
90	6.4 (5.5–7.3)	0.29 (0.14–0.30)		0 (0–20)	0 (0–36)	2

Data are presented as median and quartiles (Q1–Q3). CHO prior to exercise (from arrival at the lab) and during exercise are presented as median and (total grams), and do not include the standard Glucerna bar. HRR - heart rate reserve. *Amount of insulin taken the day of the exercise session, not including post-exercise.

**Table 4:** Change in blood glucose during and after exercise across the study arms.

Study Arm (% HRR)	Change in Blood Glucose During Exercise (mmol/L)	Change in Blood Glucose 1 Hour Post Exercise (mmol/L)
45–55%	0.9 (0.0–2.7)	−0.2 (−0.6–(−0.1))
70%	1.1 (0.2–1.6)	−0.2 (−0.9–0.2)
80%	0.7 (−0.4–3.0)	−0.1 (−0.6–0.3)
90%	1.0 (0.0–2.0)	−0.4 (−0.9–(−0.2))

Data are presented as median values and (Q1–Q3).

### Primary Outcome

The percentage of time spent ≤3.9 mmol/L in the 12 hours following exercise was not different among the three experimental conditions compared to the control condition: control [0.0 (IQR: 0.0–2.1)], 70% HRR [0.0 (IQR: 0.0–8.8)], 80% HRR [0.0 (IQR: 0.0–0.0)], and 90% HRR [16.9 (IQR: 3.3–25.7)], *p* = 0.22.

### Secondary Outcomes

**Figure 1:**
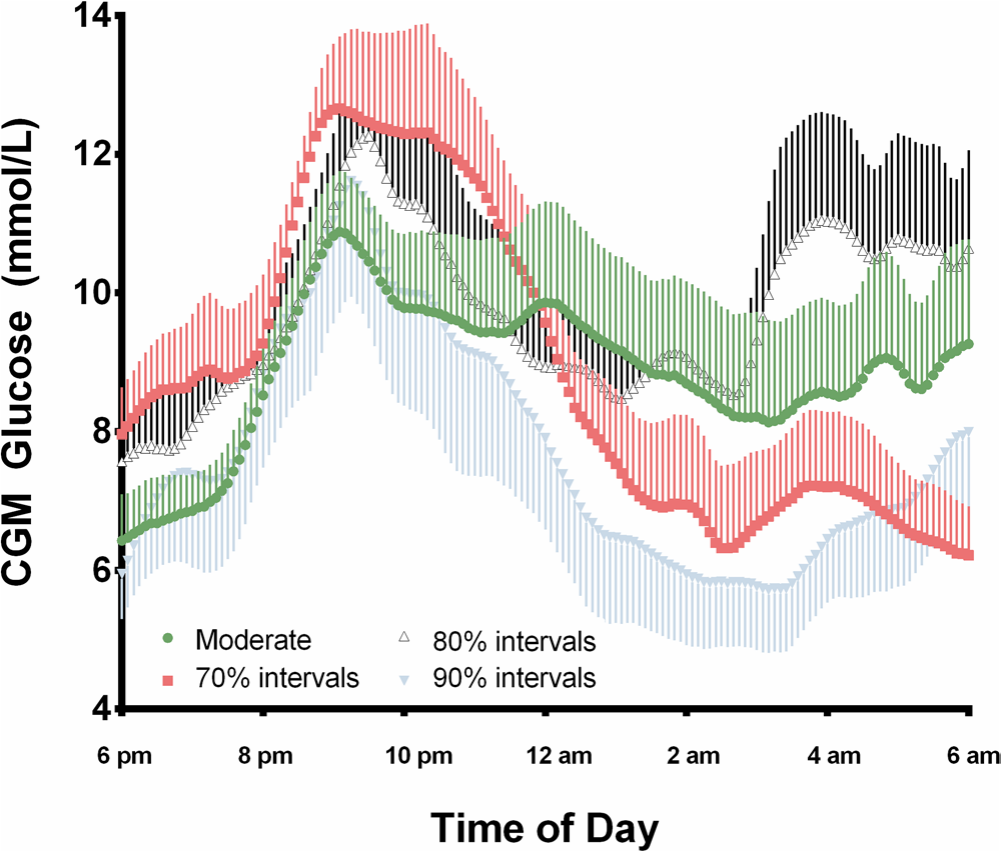
Mean interstitial glucose values in the 12 hours following exercise. Data are presented as mean values (±SEM) for all participants over the four exercise sessions in the 12 hours following exercise as measured by CGM.

Figure 1 shows mean CGM values for 12 hours following exercise in each condition. There were no differences in post-exercise mean absolute glucose (MAG) values across the four study arms. Continuous overall net glycemic action at 1, 2 and 4 hours (CONGA1, CONGA2 and CONGA4) were not significantly different across study arms (Table 5).

**Table 5:** The effect of exercise intensity on glucose variability in individuals living with T1D.

Outcome	Study ARM (%HRR)			
	45–55%	70%	80%	90%
MAG	2.9 (2.0–3.3)	2.7 (2.0–2.9)	3.2 (2.2–3.7)	2.6 (1.6–3.8)
CONGA 1	2.1 (1.3–2.6)	1.9 (1.5–2.8)	2.1 (1.8–2.3)	2.1 (1.6–3.0)
CONGA 2	3.0 (1.7–4.0)	2.0 (2.2–4.4)	2.8 (2.4–3.9)	3.4 (2.3–5.0)
CONGA 4	2.8 (2.0–3.9)	4.1 (2.5–4.6)	3.0 (2.1–5.3)	4.2 (2.7–5.6)

Data are presented as median values and (Q1–Q3). MAG = mean absolute glucose, CONGA 1, 2, 4 = continuous overall net glycemic action in the 1, 2 and 4 hours post exercise. No significant group-wise differences (*p* > 0.05).

### Exploratory Outcomes

The frequency of hypoglycemic events (Table 6) was significantly different among the conditions (p = 0.041). The frequency of hypoglycemic events (12 events) was greater following the 90% HRR intervals compared to the 80% HRR intervals (2 events, p = 0.026) and moderate intensity control session (3 events, p = 0.034). Area under the curve for hypoglycemia (p = 0.106) and hyperglycemia (p = 0.282) was not different across all four exercise conditions (Table 6). Serum cortisol declined throughout exercise, however no statistically significant differences were observed at baseline and during exercise across all four exercise conditions (*p* = 0.86). Growth hormone levels were similar among conditions at baseline (*p* = 0.83). There was a trend towards an increase in growth hormone as the intensity of exercise increased. Changes in growth hormone measured at the end of intervals (*p* = 0.06) and end of exercise (*p* = 0.08) approached significance. There were no adverse events or reported side effects observed or reported by participants.

**Table 6:** The effect of exercise intensity on hypoglycemic events and glucose area under the curve for glucose in individuals living with T1D.

Outcome	Study ARM (%HRR)			
	45–55%	70%	80%	90%
AUC ≤3.9 mmol/L	0 (0–3.5)	0 (0–25.4)	0 (0–8.5)	51.7 (0–224.9)
AUC ≥11.0 mmol/L	65.3 (0–1313.0)	674.0 (160.5–905.0)	466.0 (2.6–1069.4)	212.3 (0–987.6)
Participants w/ <4.0 mmol/L	3	4	2	7
Excursions <4.0 mMol/L	3	5	2	12^†^

Data are presented as median values and (Q1–Q3). AUC = area under the curve for interstitial glucose in the 12 hours post exercise. ^†^Significantly different from 45–55%.

## Discussion

The results of this trial do not support those of a previous experimental trial indicating that the adoption of vigorous intensity interval exercise protects against nocturnal hypoglycemia among people living with T1D^12^. Our results, however, are congruent with other studies that did not find a protective effect of vigorous intensity activity on nocturnal hypoglycemia risk^9,22,29^. While mean CGM glucose and glucose variability were not significantly different among the exercise arms, hypoglycemic events were more frequent after the highest intensity (90% HRR) interval session, relative to the other experimental and control conditions. Collectively, these findings limit the practical application of vigorous intensity interval training for stabilizing blood glucose following moderate intensity endurance exercise or preventing exercise-related nocturnal hypoglycemia in individuals with T1D.

The results of the present study are in agreement with Campbell *et al*.^9^ who did not find a difference with respect to mean nocturnal interstitial glucose and time spent in hypoglycemia post-exercise between high intensity intermittent exercise (a combination of walking, sprinting, jogging and high intensity running known as the Loughborough Intermittent Shuttle Test) and a comparable duration of treadmill running at ~77% VO_2peak_. Acutely, however, the study found a trend towards higher area under the curve (as measured by CGM) and less frequent hypoglycemia in the first hour after exercise, but these differences were not statistically significant. Similarly, Moser *et al*.^22^ found no difference in the occurrence of nocturnal hypoglycemia among three different, 30-minute vigorous intensity interval exercise protocols (maximal effort for 20 seconds interspersed with 120-second, 60-second or 20-second recovery periods respectively) and continuous moderate exercise sessions of comparable mean workloads and duration. It should be noted, however, that unlike the present study, participants were not blinded to the real-time CGM during and after these sessions, which may have decreased the likelihood of hypoglycemia as participants were aware of the level and trend in their glucose.

The elevated frequency of post-exercise hypoglycemic episodes with vigorous intensity interval exercise is consistent with a study by Maran *et al*.^29^, where a 30-minute vigorous intensity interval session consisting of 5 seconds at 85% VO_2peak_ every two minutes during a 30-minute cycling exercise session at 40% of VO_2peak_ cycling led to significantly more nocturnal hypoglycemic events (n = 7), compared to 30 minutes of moderate (40% VO_2peak_) cycling alone (n = 2, p < 0.05). In addition, the participants in this study (8 males aged 34 ± 7 years) were similar to those of the present study in being relatively sedentary (VO_2peak_ = 33.7 ± 6.1 ml/kg/min). Lower fitness levels could lead to relatively lower hormonal responses to high intensity exercise^34^ and explain the differences between these studies and others demonstrating a protective effect of vigorous intensity exercise on hypoglycemia. Collectively, these data, taken with ours, demonstrate that any protection provided by vigorous intensity interval exercise is short lived, and does not provide long-term protection against hypoglycemia among younger, less fit, individuals living with T1D.

In contrast to the data presented here, a similar trial by Iscoe and Riddell^12^ found less CGM-derived nocturnal hypoglycemia (1.5% versus 5.2%) after a vigorous intensity intermittent protocol (15-second bouts at 100% VO_2peak_ every 5 minutes during a 50% VO_2peak_ cycling session) when compared to a moderate exercise session (55% VO_2peak_ cycling for 45 minutes) matched for work output. The protective effect of vigorous intensity exercise in this study may be due to the relatively higher intensity of the intervals, or to the fact that the participants had a higher aerobic fitness level compared to those in our study and the study by Maran *et al*. (mean VO_2peak_ = 42.4 ± 1.6 ml/kg/min compared to 35.3 ± 7.6 and 33.7 ± 6.1 ml/kg/min). In contrast to untrained individuals, trained individuals are known to have greater counter-regulatory hormone responses even at moderate intensities^34^. The untrained participants in the present study may have been equally unable to take full advantage of the counter-regulatory hormone responses conferred by the high intensity intervals.

Exercise timing varied across studies of vigorous intensity exercise in individuals with T1D, with some performing sessions in the morning^9^, early afternoon^29^, or early evening^12^. Differences in how much time had elapsed between the exercise sessions and overnight monitoring, as well as how much food had been ingested prior to bed time, could affect the repletion of glycogen stores post-exercise, and consequently impact the risk of post-exercise hypoglycemia. The variation in protocols across more recent studies, including the one presented here, reinforce the concept that translating the findings from carefully controlled acute crossover trials into a practical setting may be challenging. Finally, these findings, combined with recent findings that vigorous intensity interval training may impair hypoglycemia awareness^35^, suggest that this approach for preventing hypoglycemia in those with T1D should be used with caution.

This study builds on the work of previous trials as we also examined the effects of vigorous intensity exercise on glucose variability in individuals with T1D. Increased glucose variability was associated with an increased hypoglycemia risk in the Diabetes Control and Complications Trial^19^. Higher glucose variability is also associated with fear of hypoglycemia^3^ which is known to have a negative impact on physical activity levels in people with T1D^1,3^. The only other study to examine changes in variability following vigorous intensity exercise^12^ relied on a crude surrogate of glucose variability. They found that compared to a day without exercise (2.0 ± 0.31 mmol/L), the standard deviation in glucose increased significantly following days when exercise was performed either at moderate intensity continuously (2.6 ± 0.51 mmol/L) (*p* < 0.05) or following exercise that included vigorous intensity intervals (2.8 ± 0.44 mmol/L)^12^. Similar to our study where we used more refined measures of variability (CGM data-derived MAG or CONGA), there were no differences in glucose variability between moderate and intermittent vigorous intensity sessions. More research is needed on this topic to ascertain the extent to which different types of exercise affect glucose variability in individuals living with T1D.

The proposed mechanism through which vigorous intensity exercise acutely reduces hypoglycemia risk is via the stimulation of counter-regulatory hormones^36,37^. Some studies have observed significant increases in growth hormone (GH) following vigorous intensity exercise compared to moderate exercise alone^7,10^, however this is not a universal finding^8,11^. We observed a trend towards elevated growth hormone (*p* = 0.06) following vigorous intensity intervals, but no differences in serum cortisol during or following exercise. It should be noted that the growth hormone response to exercise is higher in females than in males^38^, and lower in untrained individuals compared to trained individuals^34^. The present study is unique in that we included participants of both sexes and individuals with T1D who did not meet physical activity guidelines, according to self-report. Several hormonal responses to exercise and consequently fuel selection, are different between the sexes in individuals without diabetes^39^. There is no evidence to indicate that this is not also the case in individuals with T1D, and as such, the inclusion of both males and females may have increased the variability in the hormonal response to exercise^39^. It is also possible that the protective effects of vigorous intensity exercise on hypoglycemia risk are more pronounced in individuals who train on a regular basis. This variation in the physiological response to exercise reinforces the challenge in translating this approach into a practical setting.

The trial is strengthened by use of accurate and blinded tools to quantify hypoglycemia risk and glucose variability over a prolonged period following exercise. However, the study was restricted to only 10 participants (similar to several T1D exercise studies)^7–13,22,29^, which limits its generalisability. Second, the variability in the main outcome measures was significantly greater than anticipated, limiting our ability to detect an effect of the experimental conditions. Specifically, time spent in hypoglycemia after moderate exercise among the participants in this study compared to other exercise studies in individuals with T1D was relatively low. This may be due to patient adherence to insulin adjustment and carbohydrate intake recommendations. Finally, as the intervention was restricted to a single exercise session, the long-term impact of this approach on glycemic variability and hypoglycemia risk in the patient population remains unclear.

## Conclusion

The addition of vigorous intensity intervals to a moderate-intensity endurance exercise in the afternoon does not protect against post-exercise hypoglycemia among inactive people living with T1D. Longer-term clinical trials delivered in community or home settings with diverse samples are needed to clearly define the practical limits of this approach for preventing exercise-induced hypoglycemia in individuals living with T1D.

## Electronic supplementary material

Standard operating procedure document

Consort checklist

## Data Availability

The datasets generated and analysed during the current study are available from the corresponding author on reasonable request.
