# Supplementary material for: Vigorous Intervals and Hypoglycemia in Type 1 Diabetes: A Randomized Cross Over Trial
Source: Sci Rep. 2018 Oct 26;8:15879. doi: 10.1038/s41598-018-34342-6 (PMC6203731; doi:10.1038/s41598-018-34342-6)
Supplement: Supplementary file 1 — Standard operating procedure document [file 41598_2018_34342_MOESM1_ESM.pdf]

VIGOR Trial SOP

Table of Contents

Acute Study SOPs 4

Weight 4

Equipment 4

Procedure 4

Blood Pressure and Heart Rate 4

Equipment 4

Procedures 4

Accelerometry 5

Equipment 5

Procedure 5

Uploading Accelerometers 5

Participant Visit 7

Downloading Accelerometers 7

Changing the Battery 9

Analysis in Kinesoft 12

Heart Rate Monitor 20

Equipment 20

Procedure During Exercise Sessions 20

Procedure for Uploading Heart Rate Data 22

Procedure for Changing Recording Rate 26

CGMS 27

Patient Set Up 27

Equipment 27

Procedure 27

Prepare iPro2 27

Insert Sensor (done by a trained nurse) 27

Connect iPro2 28
Equipment 28
Procedure: 28
Remove and Clean iPro2 28
Upload Data 29
Log Sheets and Reports 29
What to do if the Patient forgot their Logbook 29
Tips for Uploading CGM 29
Guidelines for Insulin Adjustments and Glucose Supplementation 31
Insulin and Glucose Guidelines for Exercise 31
General Recommendations 32
Data Entry 32
Exercise Calibration, Testing, and Cleaning 33
Calibration 33
Testing 34
Staff Member Roles 34
Procedure 34
Cleaning 36
Maximal Exercise Test for Baseline Visit 37
Exercise Session 38
Procedure 38
Exercise Session Recording Sheet: Moderate 39
Exercise Session Recording Sheet: Vigorous 40
Important Things to Remember 40
Blood Sampling Timeline and Checklist 41
Prior to Participant Arrival 41
Supplies for Nurses 42
For Samples Drawn at 0, 10, 35, 45, 75, and 105 minutes 42
Special Note for Blood Draws 43

Updated December 10, 2014

Acute Study SOPs

Weight

Equipment

Platform scale

Procedure

Weight is measured in light clothing. Instruct participant to remove shoes prior to obtaining measurement. Ensure the scale has been calibrated using standard 25kg weights. Record weight in kilograms to the nearest 0.1kg on the case report form. Have the participant step off the scale and re-set it; perform a second measurement and record.

Blood Pressure and Heart Rate

Equipment

Validated automated BP monitor

Procedures

- Ask participant to remove tight-fitting clothing from his/her arm.
- The participant must be sitting for ≥ 5 minutes.
- Secure the cuff around the participant's left arm, making sure that the bottom edge of the cuff is approximately one-half inch (1.25cm) above the elbow, that the arrow on the scuff is in line with the brachial artery, and that the dotted line on the cuff is within the indicated zone.
- The entire cuff should be evenly tight around the participant's arm.
- The arm should rest, supported up at level of the heart
- Press ON/OFF button. Once the display panel illuminates, press the Start Button. Ask the participant to remain still until the measurement is complete.
- After the measurement is complete, the machine will display the systolic and diastolic blood pressures and pulse rate, and automatically deflates the cuff.
- Take 5 readings, at least 1-2 minutes apart and record all, including heart rate, on the case report form.

Reference: HOPE-3 Trial CRF's (L. Berard)

Accelerometry

Equipment

- Actical Accelerometer
- Accelerometer reader
- Actical software
- Kinesoft software

Procedure

Uploading Accelerometers

- Put USB from reader into the computer. Make sure the switch on the reader is on.
- Open ***Actical 3.0***
- IF using the laptop, plug the USB from the reader into the USB port labeled ***"COM4"***
- IF using the lab computer that has Actical, plug the USB from the reader into the top left USB port on the computer. This should be COM5.
- Go To Reader, Open COM port, and Select the appropriate COM Port (laptop=COM4, desktop=COM5)

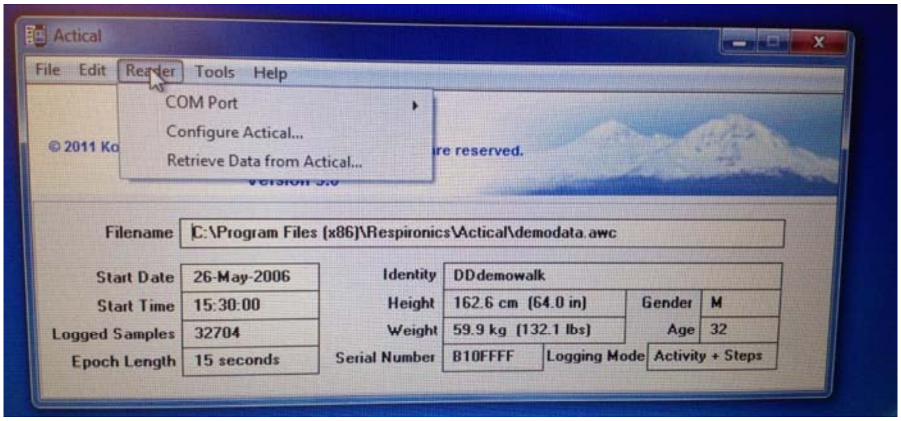

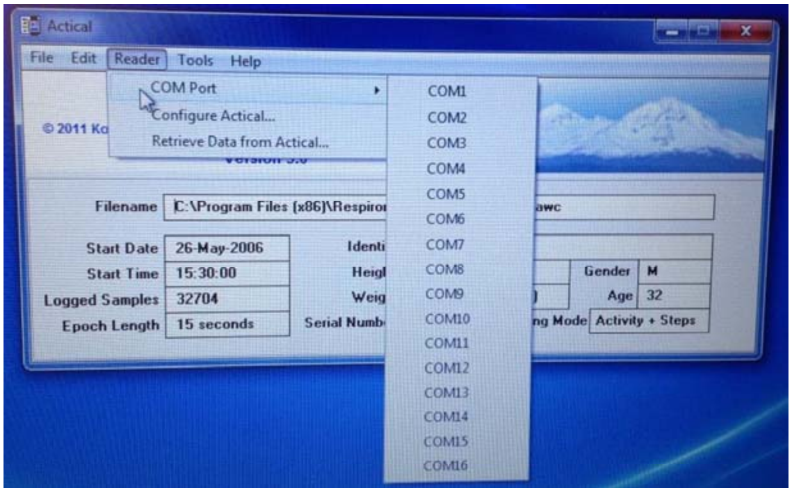

- Put accelerometer on reader. There is a green dot on the back of the accelerometer, make sure the green dot is parallel to the green dot on the reader. Accelerometer will be placed grey part down, silver part facing up.

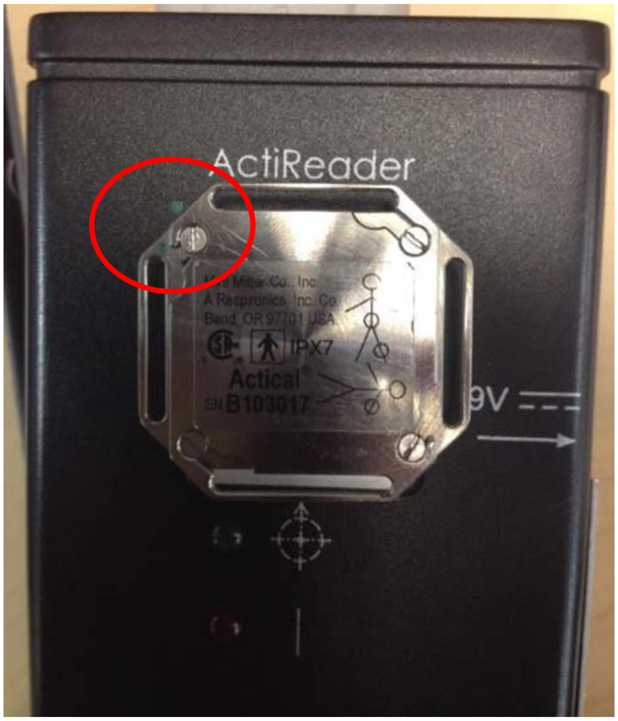

- Go to Reader, click “Configure Actical”, begin process.

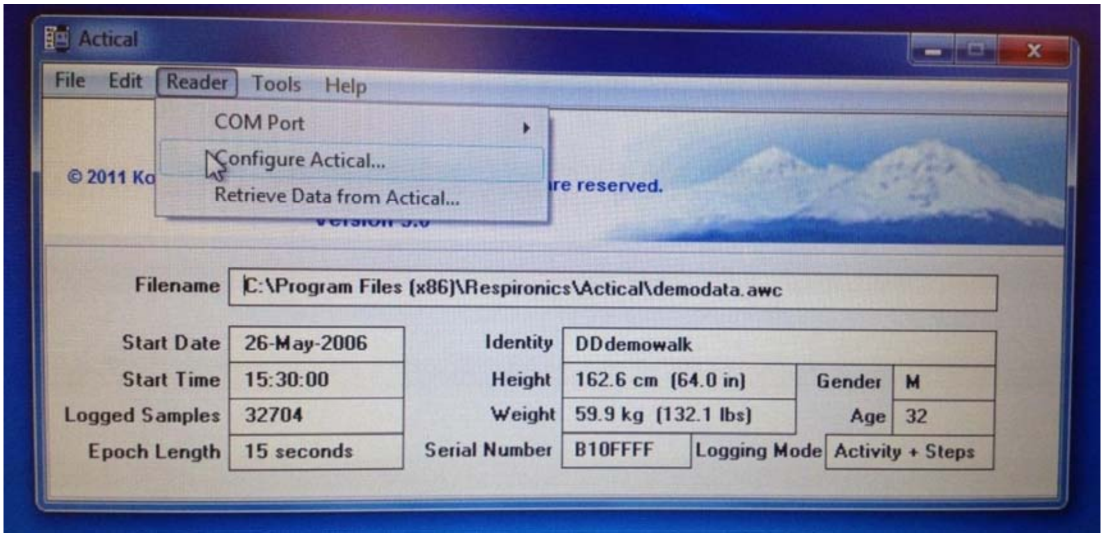

- Make sure to put accurate start time and date and check off the step box to record steps if this is desired. Put the start time as morning after the participant receives the accelerometer at 6:00 AM.
- Make sure ***epoch length is 0.50***, approximately 11 days
- Click ***Send***
- Note: accelerometer battery life will have to be monitored. It is not necessary to change battery after every participant, but in the Actical Setup screen, check the field called "Battery Life (approximate)" to make sure the accelerometer you are giving out will have enough battery to record the whole 7 days the participant will wear it.

Participant Visit

- Explain to the participant how to wear the accelerometer:
    - Place the unit on the right hip, in line with the iliac crest
    - The unit should be positioned with the word Actical right side up at the bottom of the unit
    - The belt should be adjusted so that it fits tightly around the participants waist (i.e snug)
    - Instruct participant to wear the device for 7 days, throughout their entire waking day
- Hand out logbook with accelerometer and instruct how to fill out
- Confirm details of pick-up or drop-off date when data collection is done

Downloading Accelerometers

- Put USB from reader into the laptop. Make sure switch on the reader is on.
- Open Actical 3.0
- Go To Reader, Open COM port, Select COM port displayed on pop-up message when reader initially plugged in
- Put accelerometer on reader. There is a green dot on the back of the accelerometer, make sure the green dot is parallel to the green dot on the reader. Accelerometer will be placed, grey part down, silver part facing up (refer to picture above).
- Go to Reader, click on ***Retrieve Data from Actical*** , and begin process, then click ***continue.***

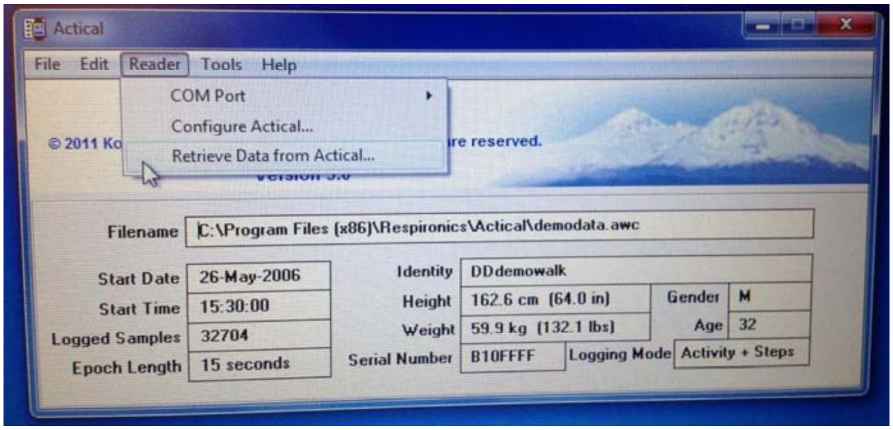

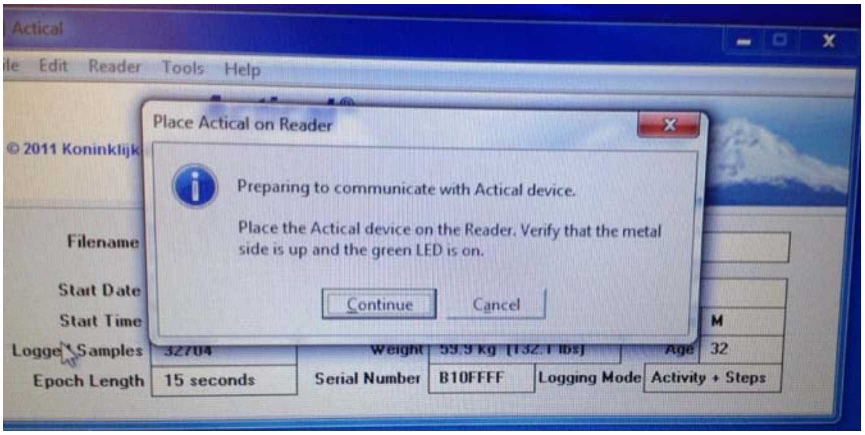

- This is what the screen will look like when the data is being uploaded:

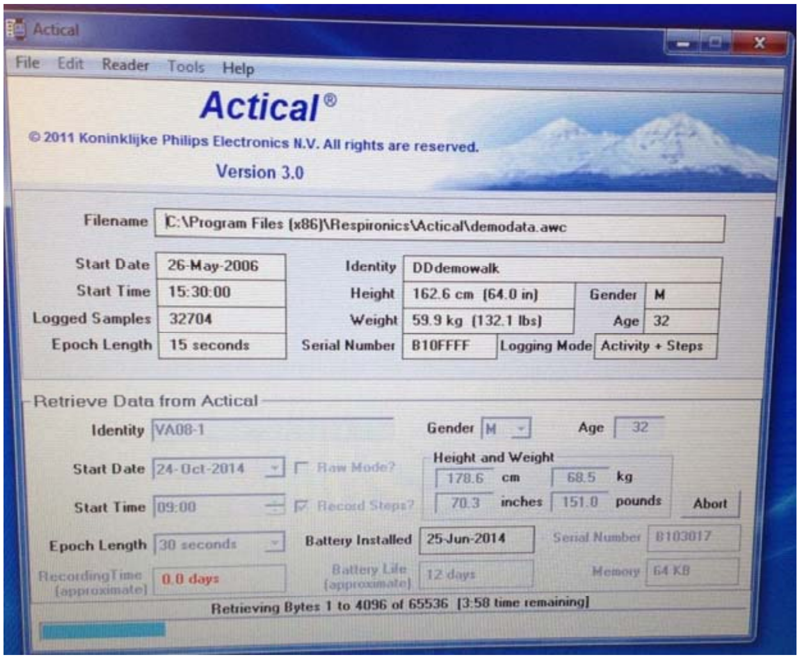

- When saving data to the computer, make sure it is saved in the VIGOR data folder under the subjects ID

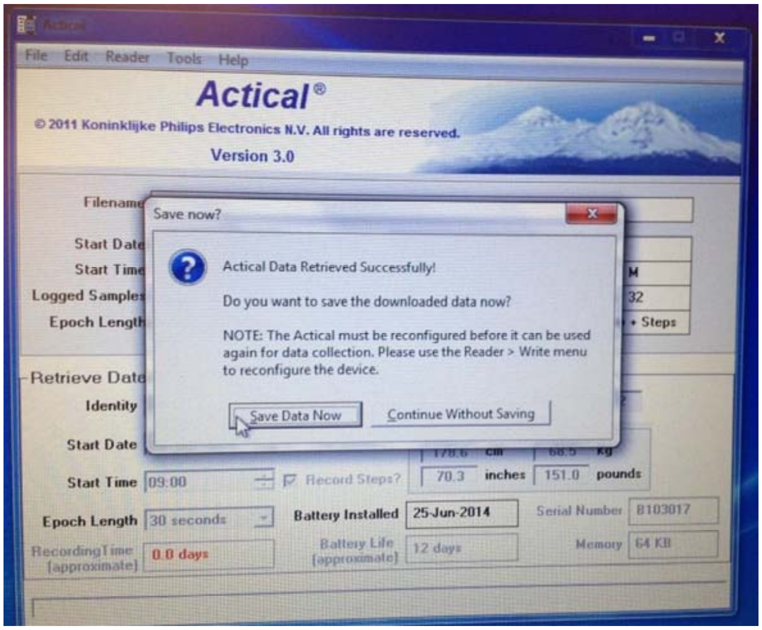

Changing the Battery

- When Actical gives a warning that battery life is low, it is important to change the battery.
- To replace the batter unscrew the screws on the back and twist the top piece clockwise. It is important to unscrew the screws only partially to avoid losing small parts as shown in the following picture:

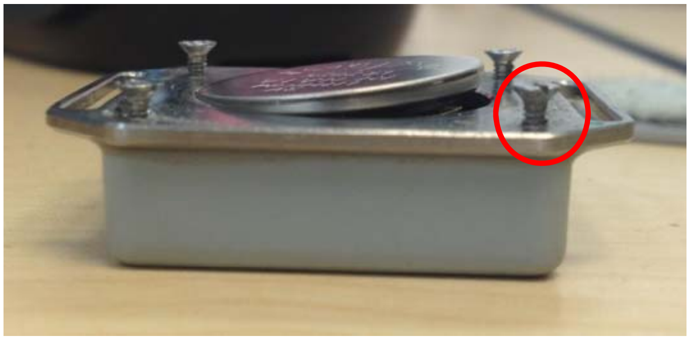

- The two pieces of the Actical are shown as follows:

Battery

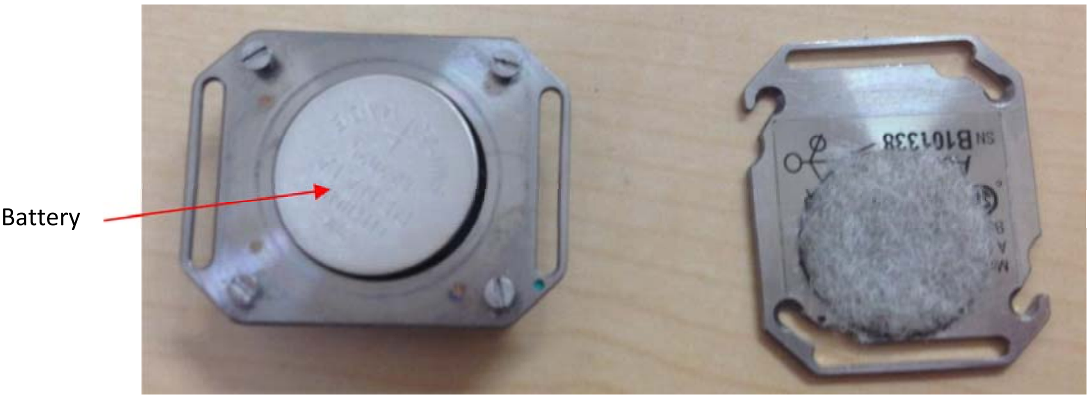

- Replace the new battery into the space indicated above with the words on the battery facing outward.
- Re-screw the backpiece onto the main Actical and place the Actical on the *ActiReader*
- In *Actcial 3.0* click on ***Reader*** and ***Configure Actical***

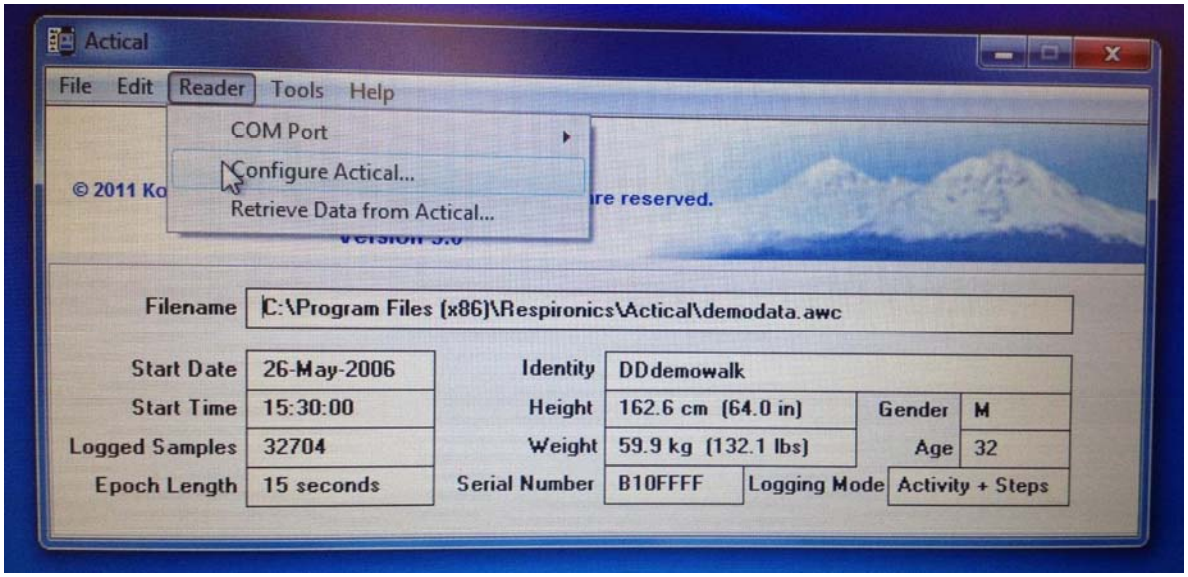

- Once the screen is loaded click on the box ***Battery Installed*.** When prompted click ***Change Date***

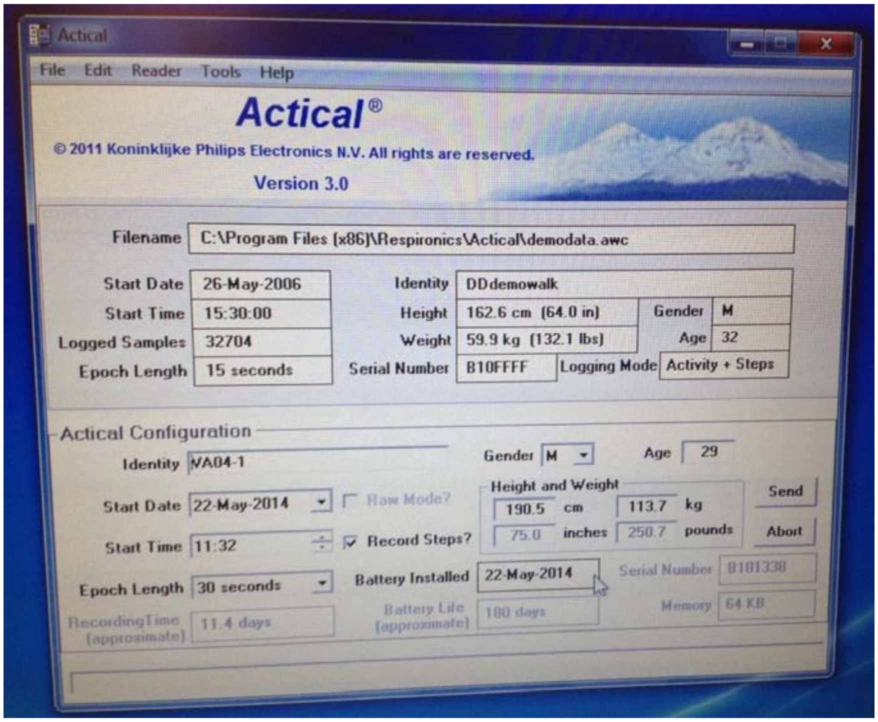

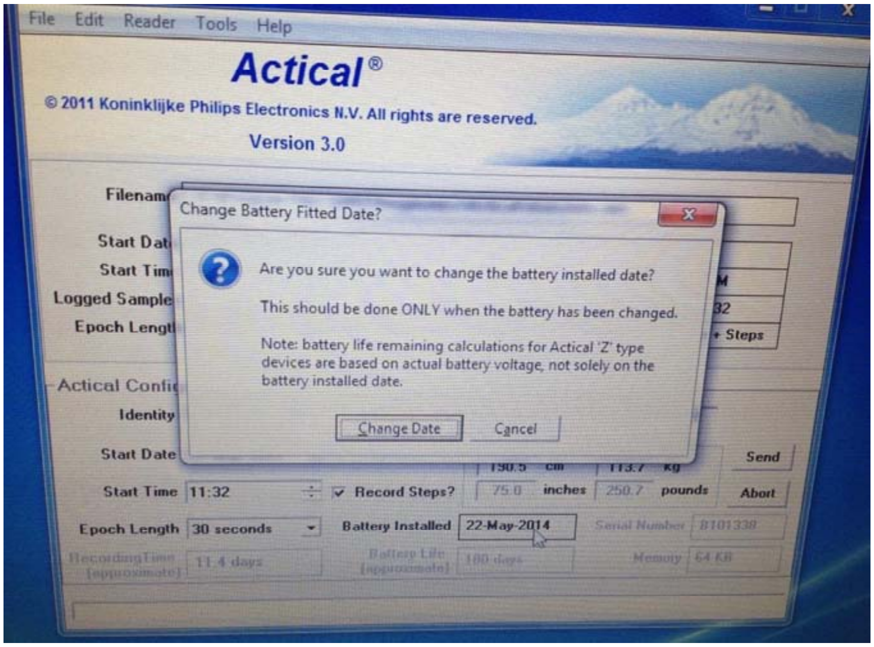

- Once ***Change Date*** is selected the current date will appear in the ***Battery Installed*** box. Then click ***Send***. Once the information is sent to the Actical the ***Recording Time*** box will change to **11.4 days**.

Analysis in Kinesoft

- Open Kinesoft software

How to Change the Settings Prior to Uploading Data

- Before uploading the accelerometer data make sure the appropriate settings are selected.
- Click on **File Settings**, then follow options below:

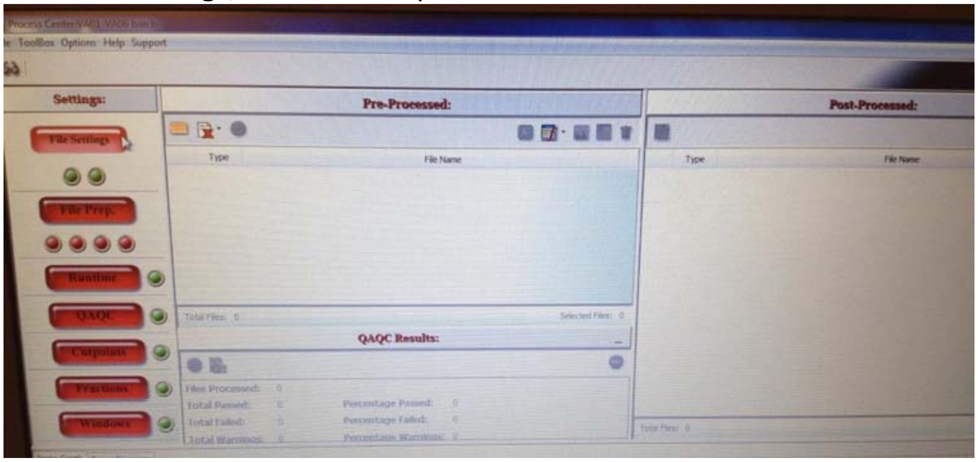

  - ***Expected epoch setting (in seconds):*** 30
  - ***Start collecting data on day:*** *1*
  - ***Start collecting data @:*** *06:00:00*
    - If the start time on the accelerometer was changed to a later time such as 9:30 AM when it was configured, 9:30 AM must be selected as the start time in this box
  - ***Number of days to collect:*** *7*

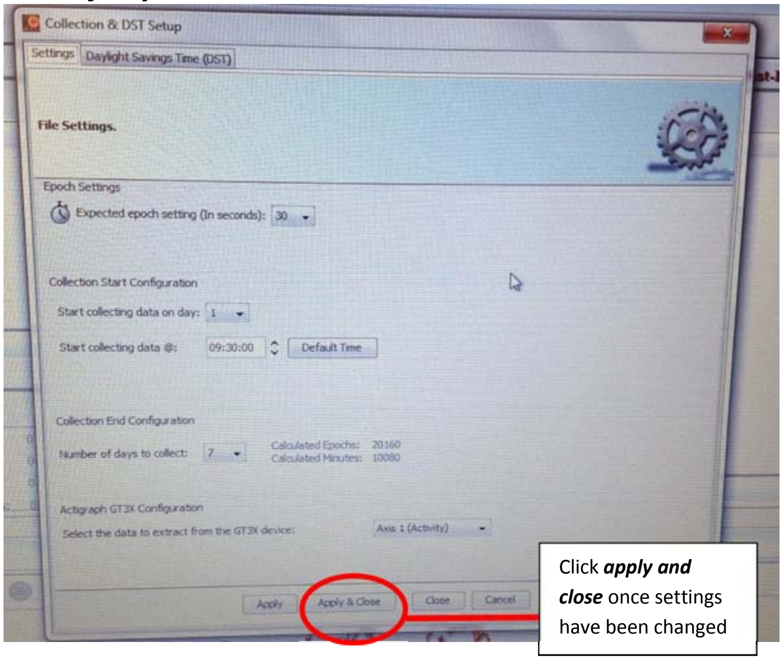

- There is nothing to change in ***File Prep*** or ***Runtime***
- Click on ***QAQC –*** the valid day criteria is based on the Canadian Health Measures Survey 2011
    - ***Valid Day Criteria:***
        - ***Minutes:*** *600*
        - ***Hours:*** *10:00*
        - ***% of Day:*** *41.66*
    - ***Valid File Criteria:***
        - ***Valid days to pass QAQC:*** *4*

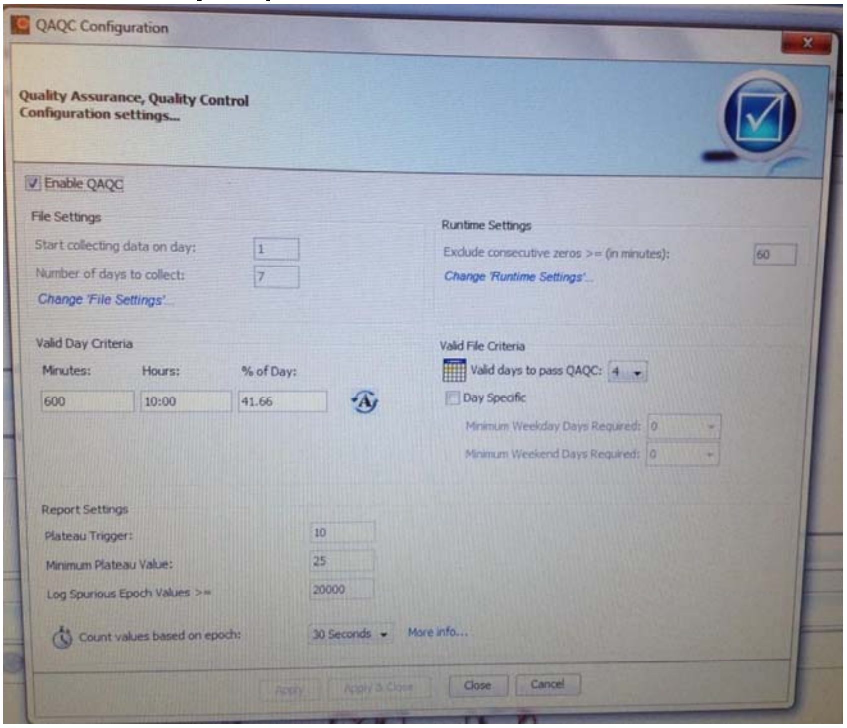

- Click on ***Cutpoints*** – the cutpoints used to define sedentary, light, moderate, vigorous and MVPA are based on the Canadian Health Measures Survey 2011
    - If the cutpoints have not been defined, these boxes will be blank. To add in cutpoints click on the white empty box under ***Cutpoint Title*** and add the title you desire (Sedentary, Light, Moderate, Vigorous, or MVPA). ***Start Value Type and End Value Type*** should be labeled as ***count***.
    - Use the following cutpoints:

Cutpoint Title	Start Value (>=)	Start Value Type	End Value (<)	End Value Type
**Sedentary**	0.0	Count	100.0	Count
**Light**	100.0	Count	1535.0	Count
**Moderate**	1535.0	Count	3962.0	Count
**Vigorous**	3962.0	Count	49999.5	Count
**MVPA**	1535.0	Count	49999.5	Count

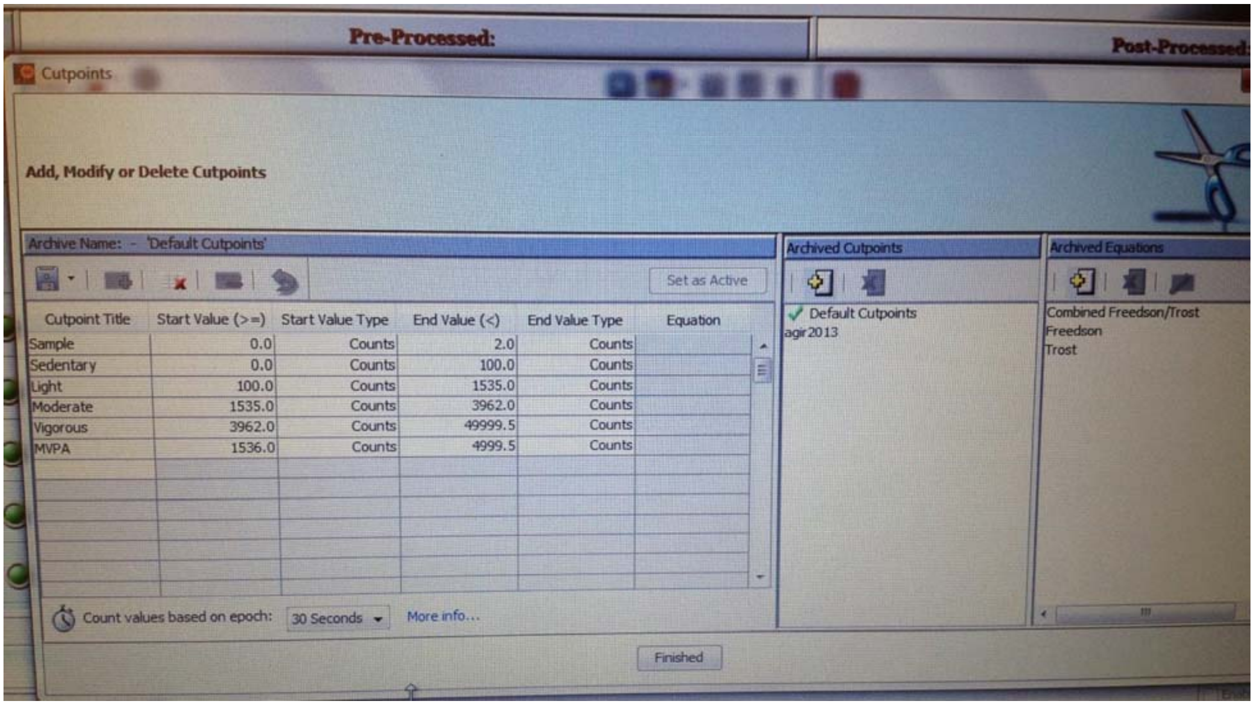

- Use the default settings under ***Fractions***
- Use the default settings under ***Windows***

Uploading Data Once Appropriate Settings have been Selected

- Now that the settings have been changed, the data can be uploaded
- Under the column marked ***Pre-Processed*** click on the *file folder.* Select the accelerometer files that are saved as an .AWC file (you can select multiple, this allows for analysis of all accelerometer files at one time). Once the files have been selected, click ***Open.***

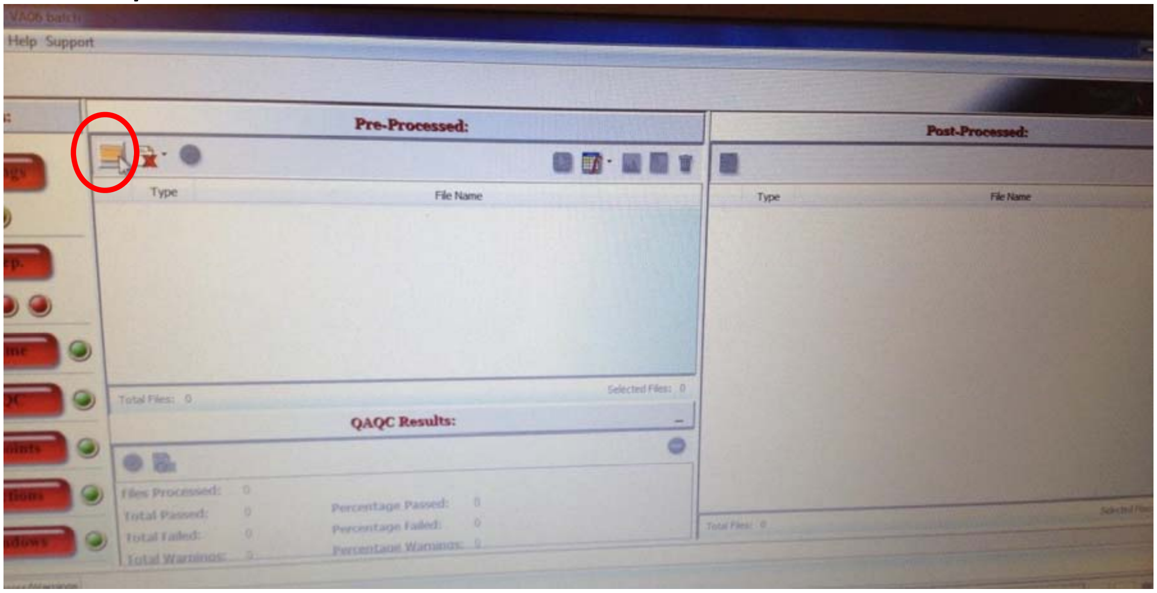

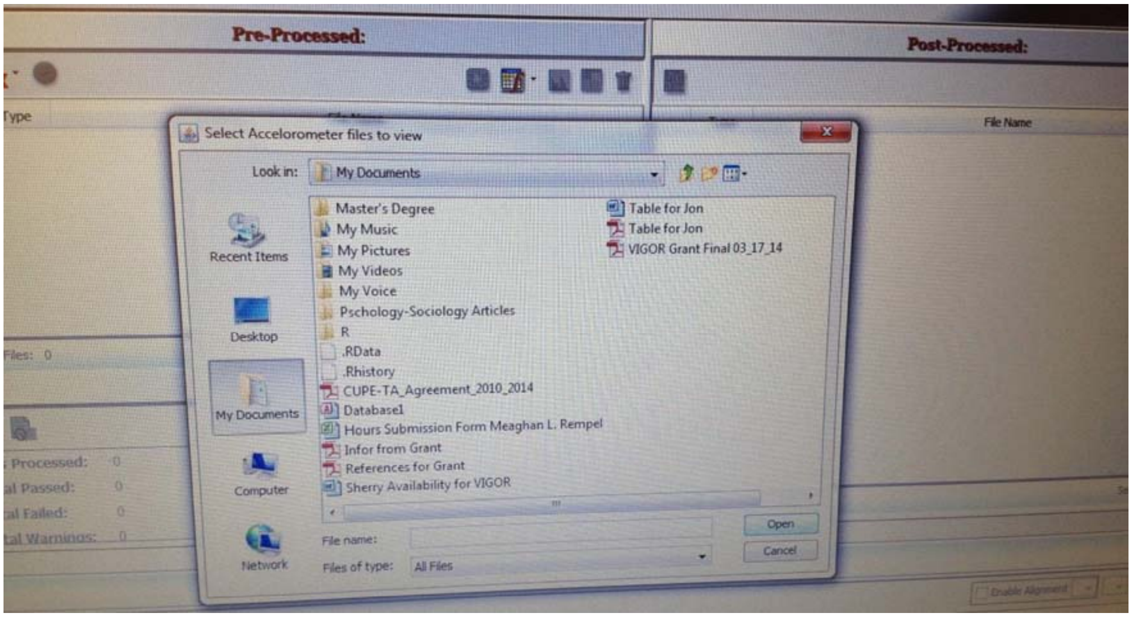
Pre-Processed:
Post-Processed:
Select Accelorometer files to view
Look in: My Documents
Recent Items
Desktop
My Documents
Computer
Network
Master's Degree
My Music
My Pictures
My Videos
My Voice
Pschology-Sociology Articles
R
.RData
.Rhistory
CUPE-TA_Agreement_2010_2014
Database1
Hours Submission Form Meaghan L. Rempel
Infor from Grant
References for Grant
Sherry Availability for VIGOR
Table for Jon
Table for Jon
VIGOR Grant Final 03_17_14
File name:
Files of type: All Files
Open
Cancel
File Name

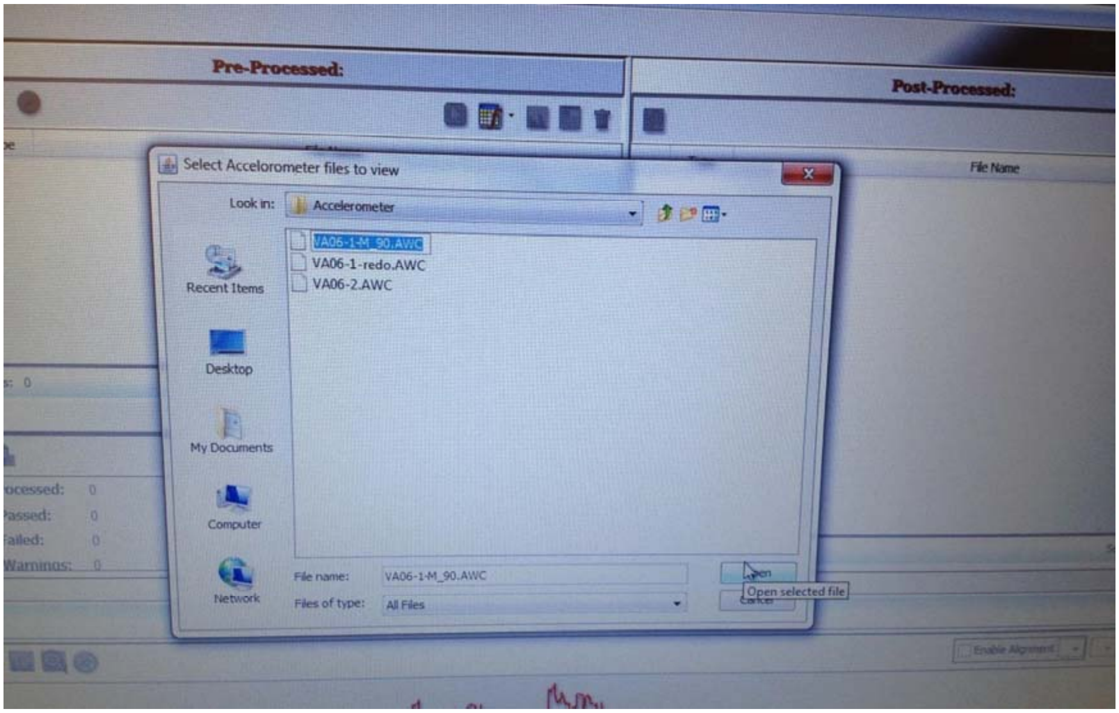
Pre-Processed:
Post-Processed:
Select Accelorometer files to view
Look in: Accelerometer
Recent Items
Desktop
My Documents
Computer
Network
VA06-1-M_90.AWC
VA06-1-redo.AWC
VA06-2.AWC
File name: VA06-1-M_90.AWC
Files of type: All Files
Open selected file
File Name

- The file will show up under the ***Pre-Processed*** column
- To run the file click on the button that looks like a green circle with a red arrow in the centre.

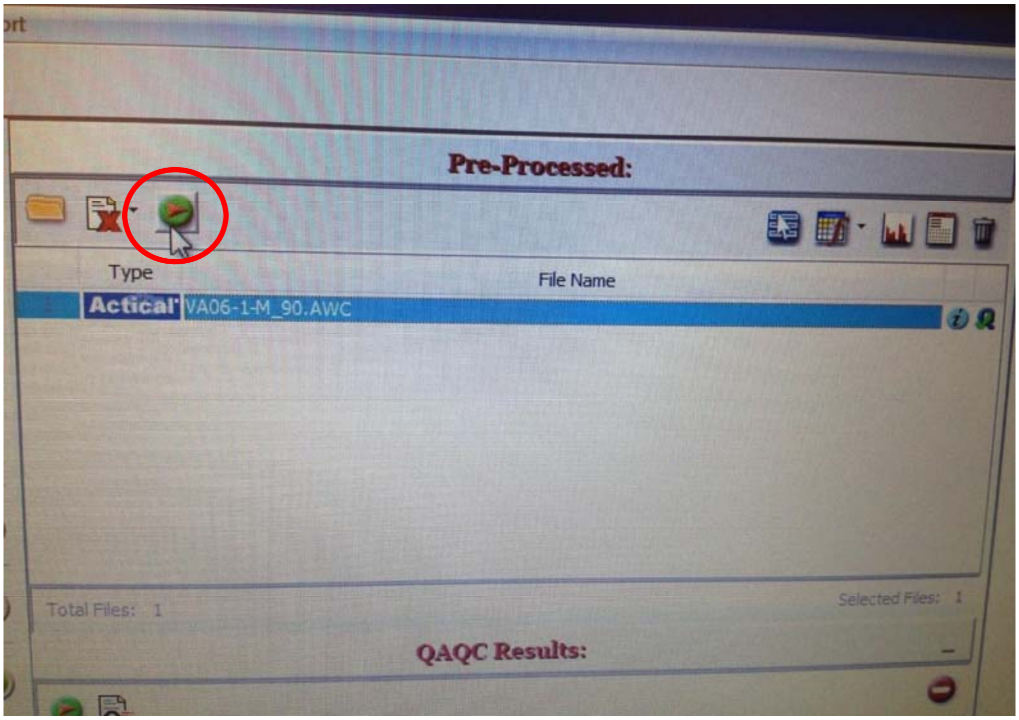

- The selected file will now appear in the ***Post-Processed*** column
- Select the file and click on the button in the top left corner

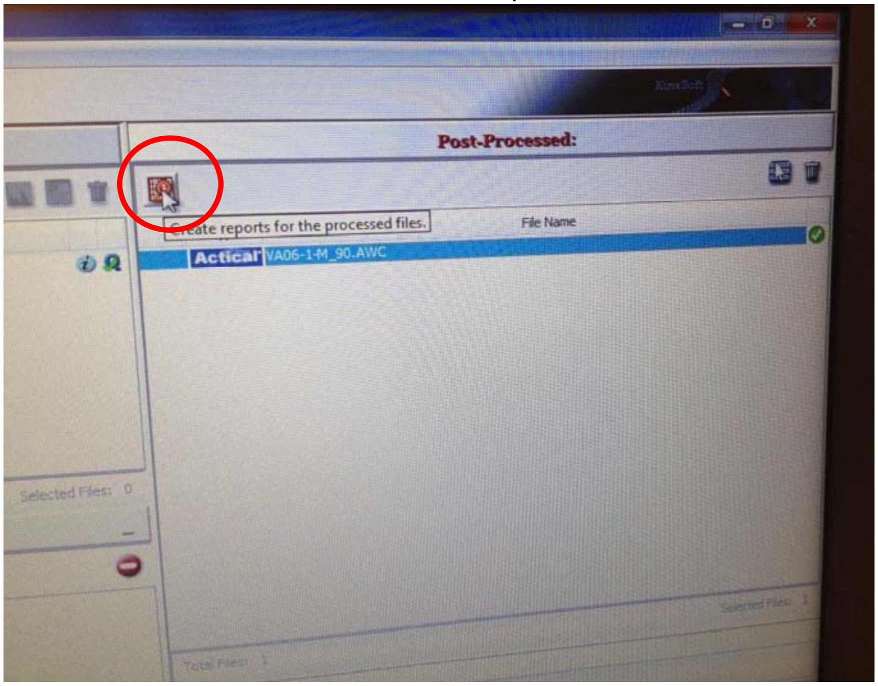

- A new screen will appear called ***Report Configuration Options***
  - Select all boxes under ***Sums and Ratios***
  - Use the default settings under ***QAQC*** (all values will be labeled as 10)

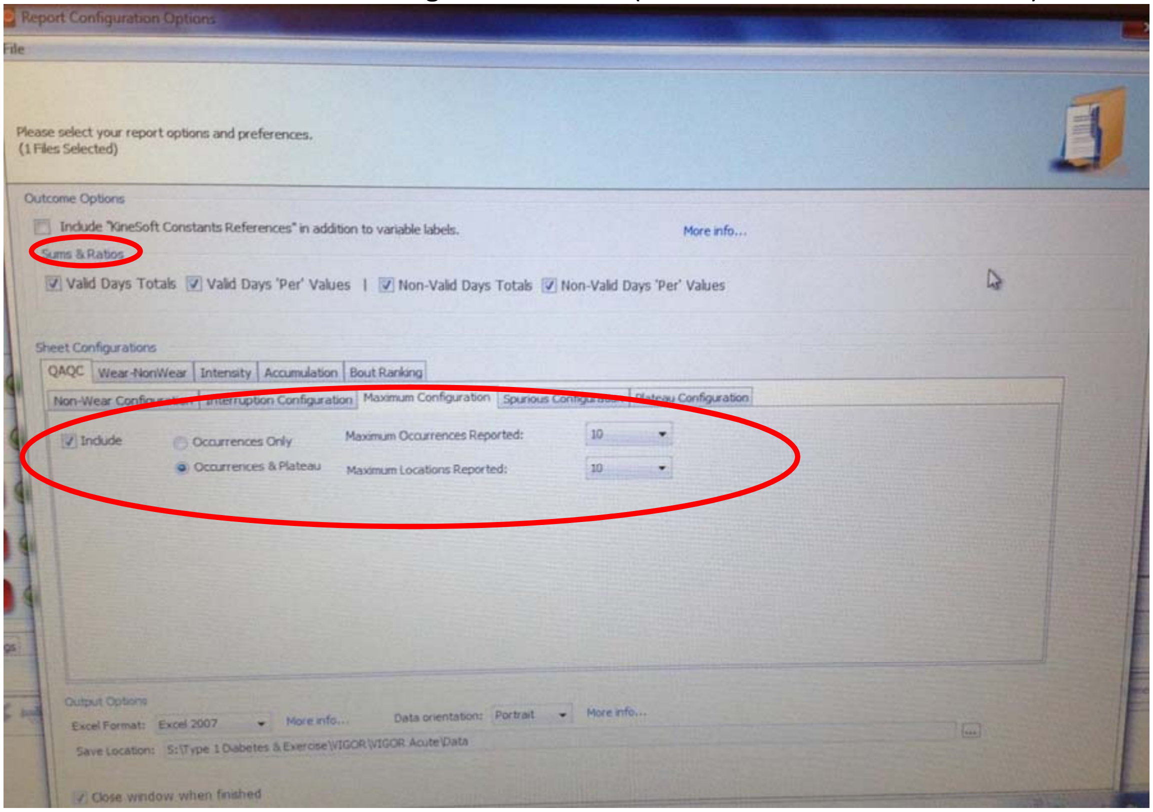

  - Click ***Wear-NonWear*** and make sure every box is selected except for the (minutes, bouts, counts, steps).

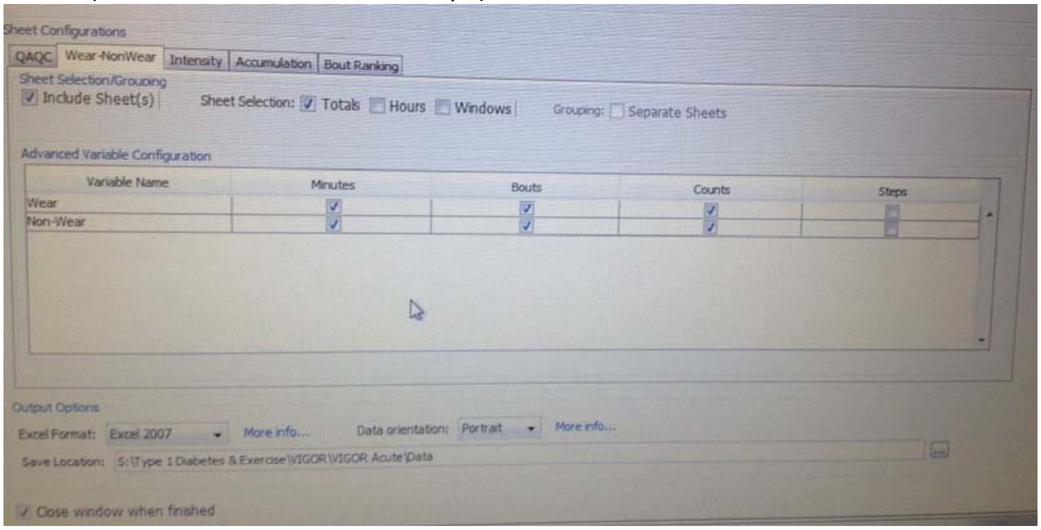

- Under ***Intensity*** and ***Accumulation*** make sure Cutpoint titles appear (Sedentary, Light, Moderate, Vigorous, MVPA). Select all boxes (minutes, bouts, counts, steps).

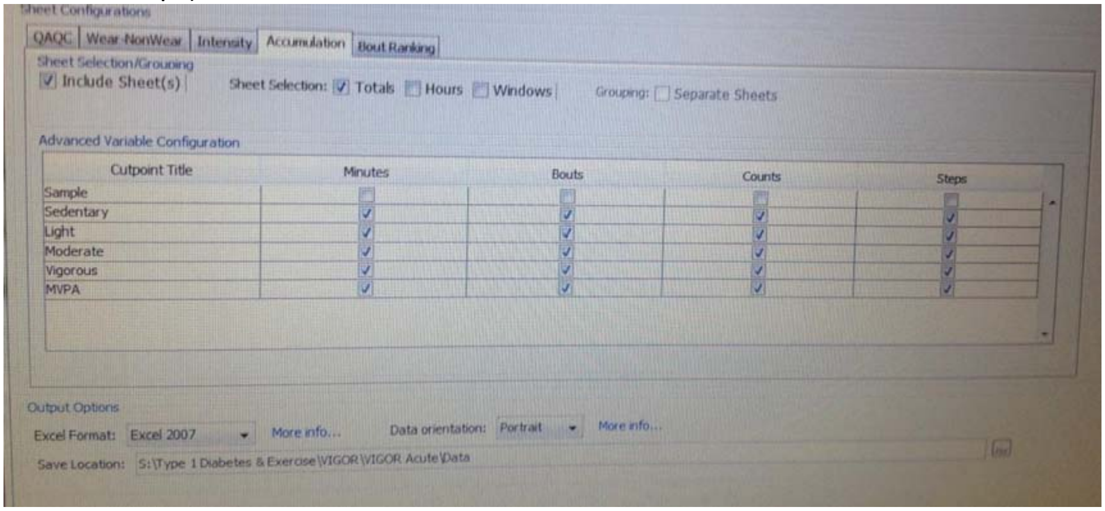

- Under ***Bout Ranking*** select all boxes (Date/Time Stamp, Minutes Only, Date Only, Time Only, Depth 200).

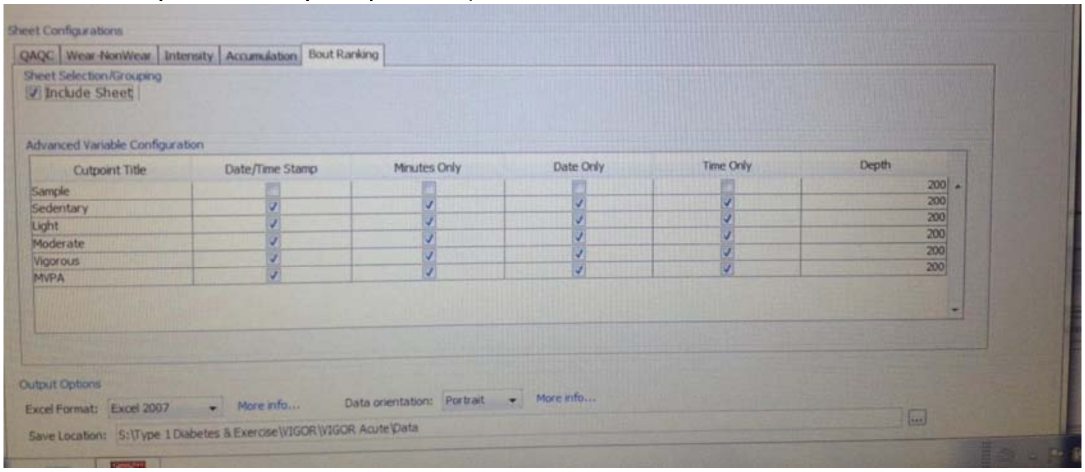

- Under ***Output Option*** make sure to select the appropriate file that the data will be saved in (it is an Excel File).
- Click on ***Generate Reports***
- The report will look something like this:

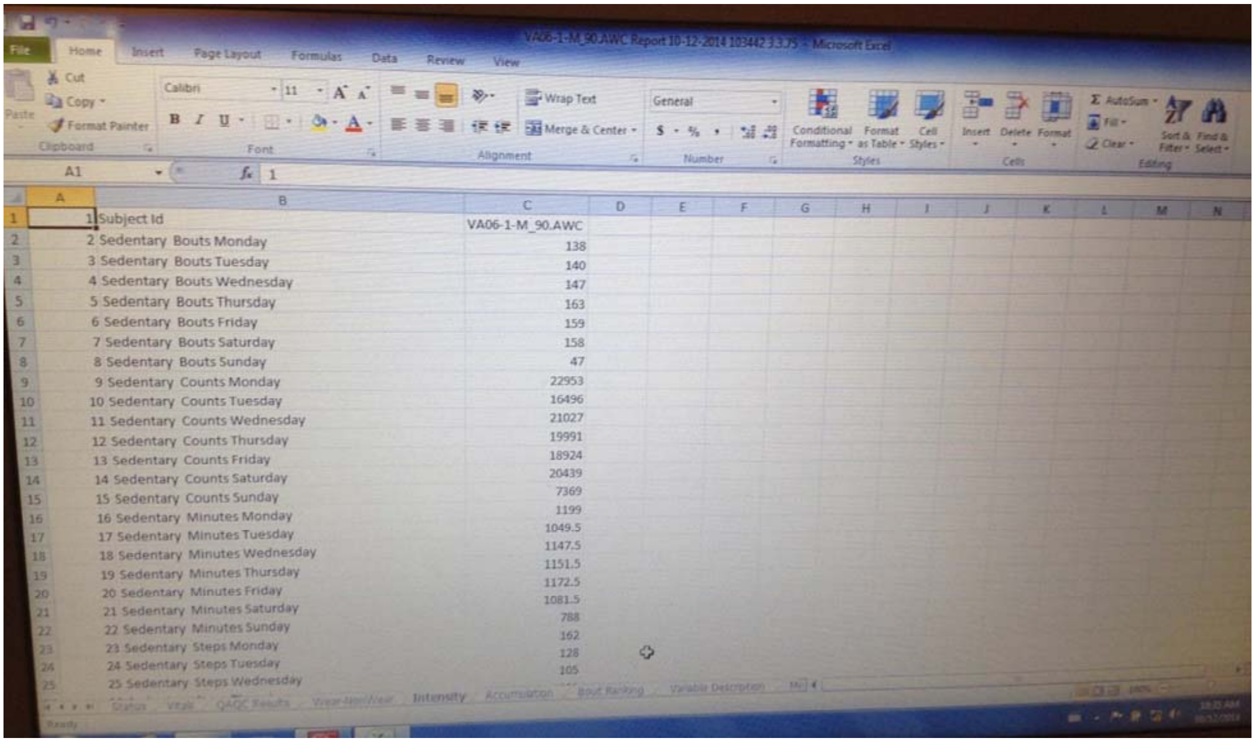

Heart Rate Monitor

Equipment

- Polar RS800CX N
    - Watch
    - WearLink®W.I.N.D.Transmitter (Connector and Strap)
    - USB transmitter

Procedure During Exercise Sessions

- Fit participant with heart monitor strap and connector

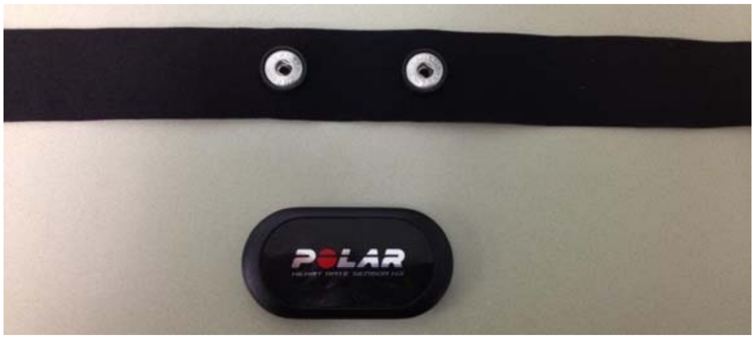

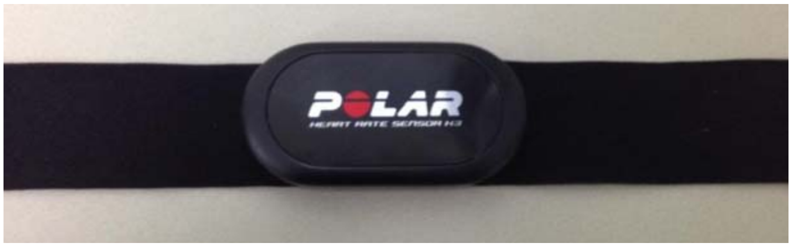

- On the watch press START

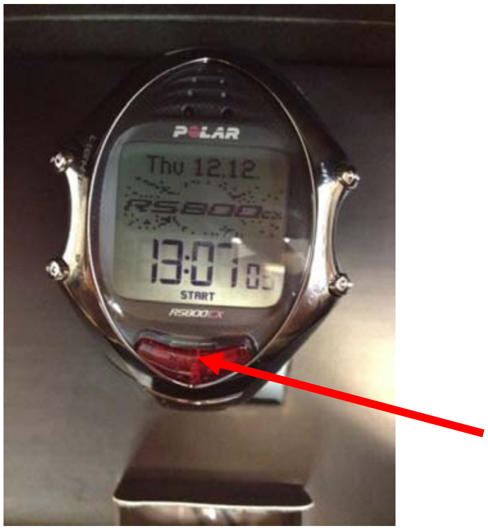

- Ensure heart rate is picked up on the watch

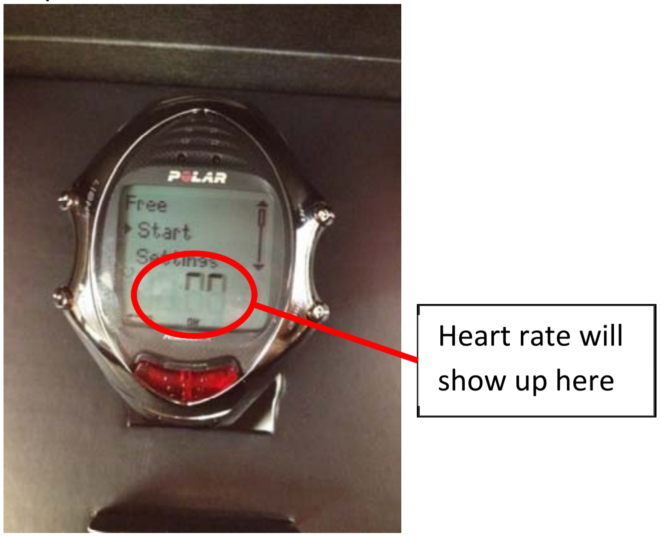

- Once the participant is reading for exercise, have them stand on the treadmill. Adjust the treadmill speed and grade to the correct intensity calculated for the participant. As soon as the participant is at the calculated intensity press **START.**

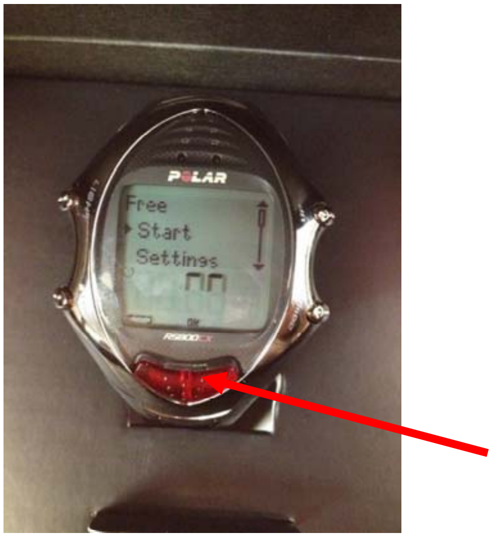

Procedure for Uploading Heart Rate Data

- Put USB transmitter into the USB port of the computer
- Unsure that the watch is close to the USB transmitter

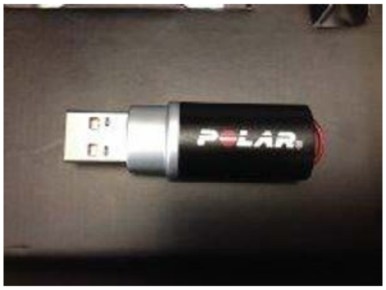

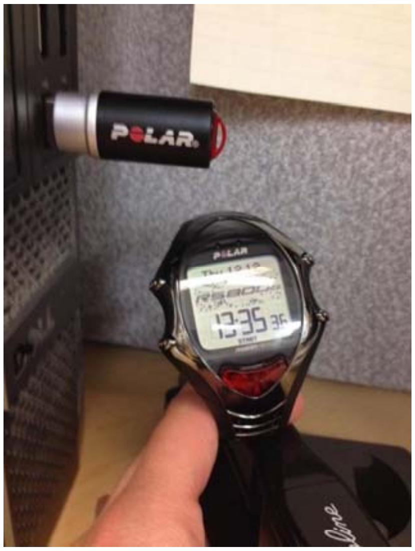

- On the watch, press DOWN and the following screen should be displayed

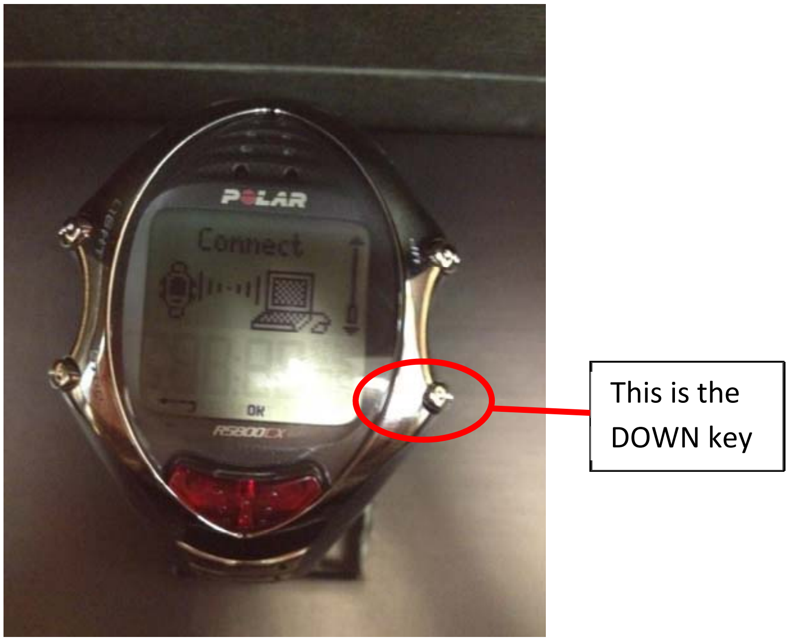

- In Polar Trainer 5 Software click on TOOLS and then TRANSFER DATA. Then select OK.

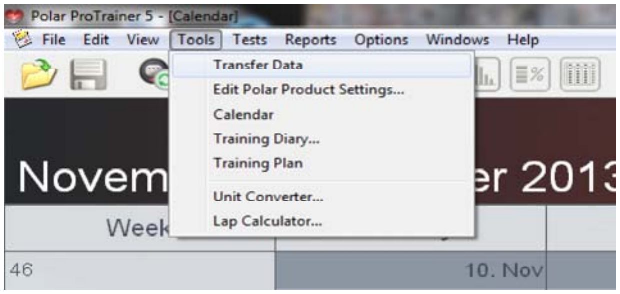

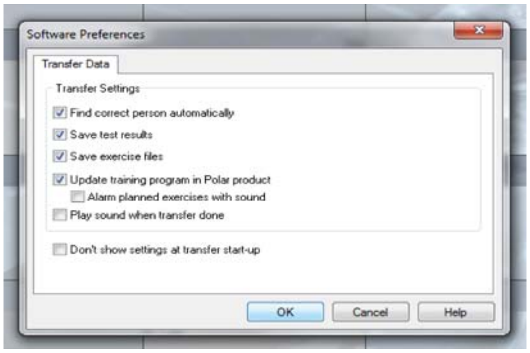

- The following screen should pop on the computer to ensure that the transfer of heart rate data is being made.

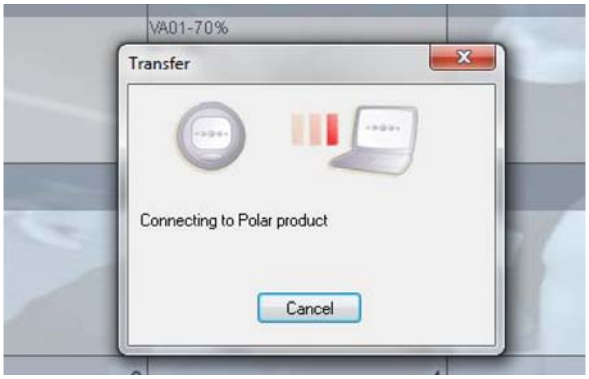

- The heart rate data will show up as FREE on the day the participant completed the session.

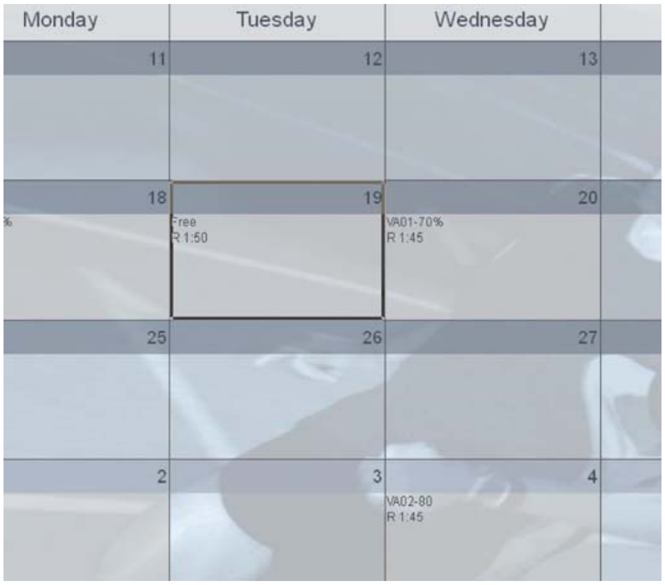

- Double clicking on the desired day will bring up the following screen:

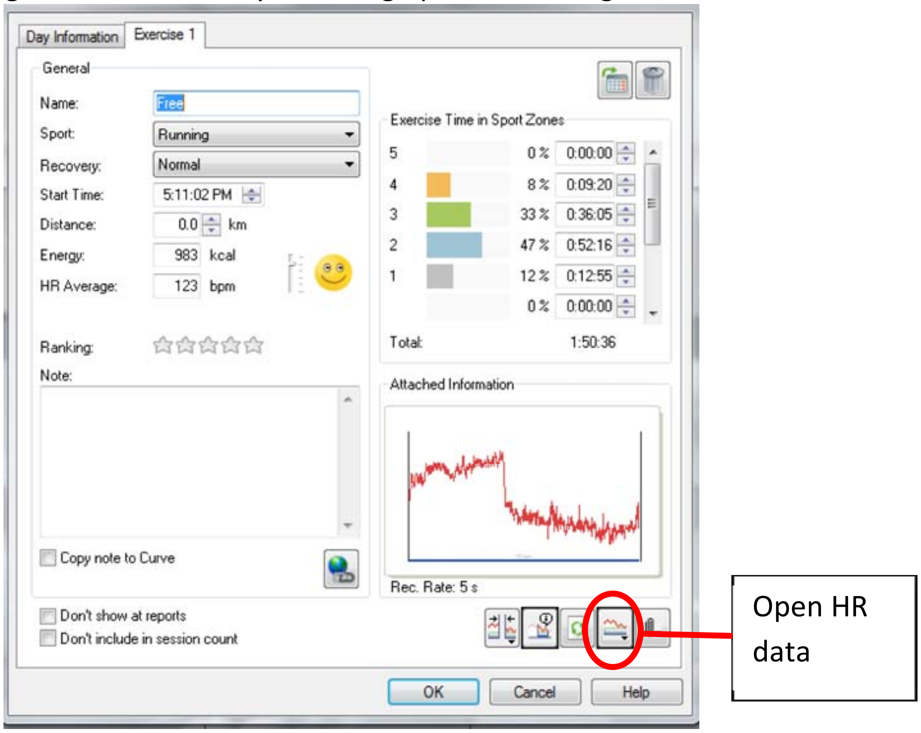

- From this screen you can change the NAME. It can be helpful to add in the participant ID and session (Example: VA02-80 – this indicates the participant ID and heart rate data for the 80% session). You can open the participants heart rate data by selecting the area circled in red.
- Once this heart data has been selected the following screen will open:

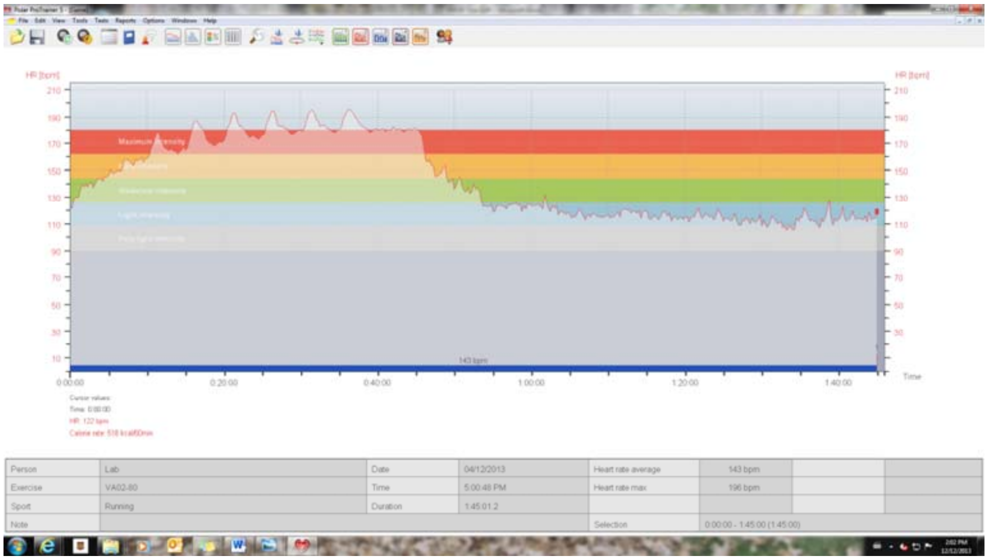

- To export data, select FILE and EXPORT AS TEXT. Then save the file in a designated folder.

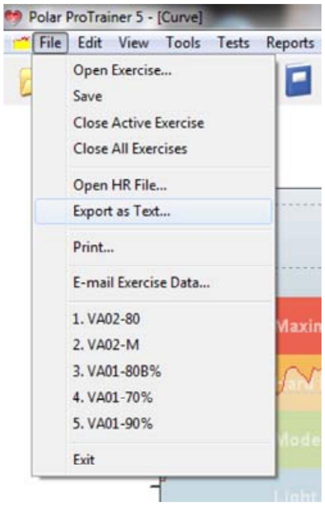

Procedure for Changing Recording Rate

- The watch can be programed to record heart rate every 5 seconds or every 15 seconds. To select the desired recording rate, press DOWN three times to see the SETTING screen. Click OK (big red button). Select FEATURES and scroll down to RECRATE 15 (this indicates the recording rate is every 15 seconds. To change this select OK and then select 5 seconds as the recording rate.

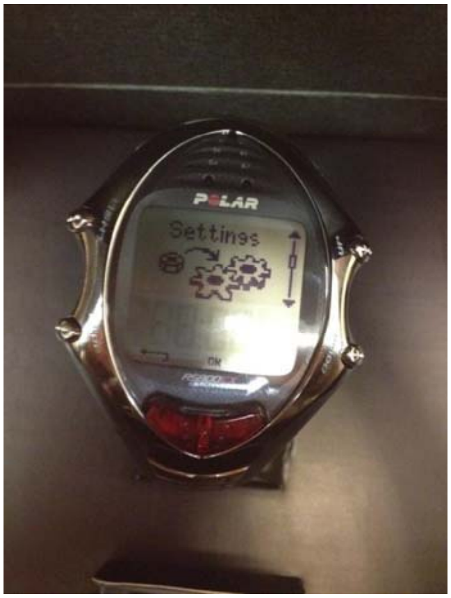

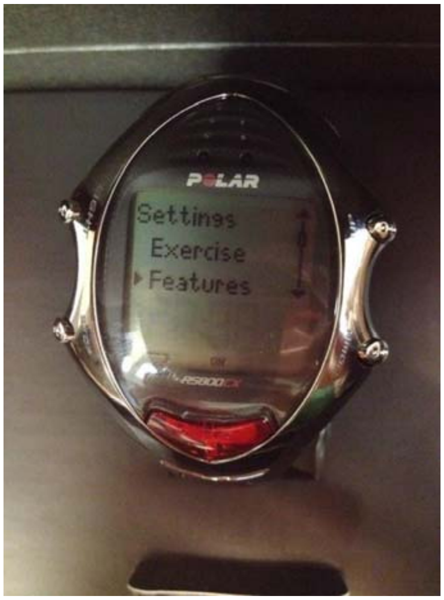

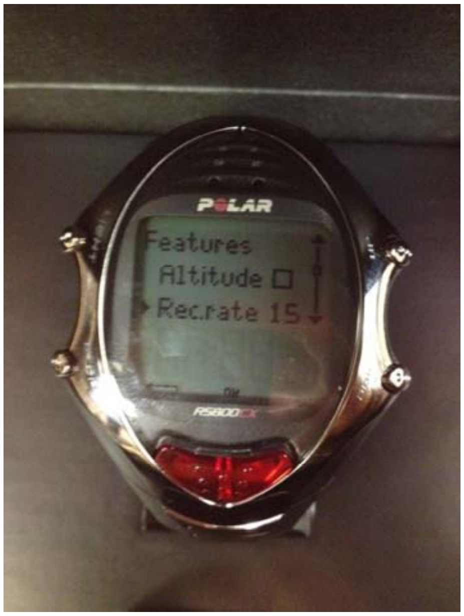

CGMS

Note: More information can be found in the iPro user manual located with all the CGM supplies, and as a .pdf on the diabetes drive.

- Be sure to ask participants if they are sick or getting sick because:
    - In can affect glucose readings
    - They may be taking cold medication which typically has glucose as an ingredient
- Be sure to tell participants to test when glucose levels are the most stable (**before** meals)

Patient Set Up

Equipment

- Gloves
- Alcohol swabs
- Sen-serter
- Glucose sensor
- Sharps container
- iPro2
- Skin prep (optional)

Procedure

Ensure the date and time on the participant's glucometer is correct to the actual date and time

Prepare iPro2

- Verify iPro2 is ready to use. Check for solid green charging light on Dock. Flashing green charging light may mean:
    - iPro2 contains participant data and needs to be uploaded before it can be used on another participant, or
    - iPro2 needs to finish charging before it can be used.
- Clean iPro2:
    - Remove iPro2 from the Dock and connect a cleaning plug.
    - Wipe iPro2 with alcohol swab. Disconnect cleaning plug.

Insert Sensor (done by a trained nurse)

- Wash hands and put on gloves.
- Select insertion site. For consistency, usually aim to insert on the back of the hip, above the buttocks.
- Clean insertion site with alcohol.
- Insert sensor using Sen-serter.
- Hold sensor in place while gently removing introducer needle. Dispose in sharps container.
- Wait 15 minutes before connecting the iPro2.

- Enter the iPro2 serial number and blood glucose meter ID on the Clinic Equipment Log and on the Patient Log Sheet.
- Train patient using Patient Instructions sheet in the patient logbook

Connect iPro2

Caution: If you see body fluid on the metal sensor contacts or black o-rings, do not connect the iPro2. Remove and dispose of the sensor, and insert a new sensor. This will prevent contamination of the iPro2.

- Connect iPro2 to sensor. Avoid twisting.
- Verify that iPro2 flashes briefly. If iPro2 does not flash within 10 seconds, disconnect from sensor and try again in 30minutes.

Uploading to CareLink™ iPro

Equipment

- Gloves
- iPro2 (which has been worn by the participant)
- Cleaning plug
- Optional: adhesive remover, such as Detachol®
- Mild liquid soap
- Quaternary ammonium compound, such as Cavicide®
- 70% isopropyl alcohol
- Bio-waste container
- Clinic Equipment Log
- Dock, connected to a computer
- Patient's blood glucose (BG) meter
- Patient Log Sheet
- Meter manufacturer's cable

Procedure:

Remove and Clean iPro2

- Wash hands and put on gloves.
- Remove iPro2 from sensor. Avoid twisting.
- Remove sensor from participant's body and dispose in bio-waste container.
- Always clean and disinfect the iPro2 before connecting it to the Dock.
- Connect a cleaning plug to the iPro2.
- Remove adhesive residue using adhesive remover (Detachol).
- Wipe with mild liquid soap solution.
- Rinse with water.
- Apply quaternary ammonium compound disinfectant (Cavicide).
- Wipe with 70% isopropyl alcohol.
- Disconnect cleaning plug and allow iPro2 to air dry.

Warning: If body fluid enters the iPro2's connector, you must dispose of the iPro2. Do not connect it to the Dock.

Upload Data

On Computer (use laptop to download data and plug it in to the network cable):

- Find the participant's record in CareLink iPro (http://ipro.medtronic.com) or create a new participant record if needed.
- Use the Clinic Equipment Log or Patient Log Sheet to identify the correct iPro2 for the patient.
- Click Upload iPro2. Follow on-screen instructions for uploading data from iPro2.
- Use the Clinic Equipment Log or Patient Log Sheet to identify the correct BG meter for the participant.
- If BG meter is supported by CareLink iPro, follow on-screen instructions for uploading data from meter.

Log Sheets and Reports

- Click **Open Logbook** to add event markers or BG meter readings from Patient Log Sheets.
- Click individual reports to view them, or click **Print all** to print them.
- If an uploaded BG meter reading should not be used for calibration of sensor data, click **Open Logbook** and then click the **Exclude** check box to exclude it.

What to do if the Patient forgot their Logbook

- If you are concerned about losing data or the battery life on the CGM you can choose from the following options:
    - Upload the data to the server without the logbook information and then add in the calibration points once the logbook has been returned
    - Plug in the CGM to a POWERED dock to prevent loss of battery life. The green battery light will flash even when fully charged to tell you that there is still data left on the CGM.
    - *Information provided by Medtronic Rep.*

Tips for Uploading CGM

- A minimum of 4 calibration points is required in order for output to be accurate. There must be calibration points in the **morning** and **before bed**.
- Some calibration points may need to be excluded for accurate output:
    - 4 – 7 mmol/L is considered normal physiologic condition for blood glucose.
        - If there is debate between two points as to whether or not it should be included or excluded, pick the point that is closer to the normal range
    - There should be no more than 8 hours between measurements
- Remember that the CGM can only read between 2.2-22.2 mmol/L

- Daily summary/overlay outputs with flat lines indicate the inability of the CGM to read above or below that value (refer to graph below)

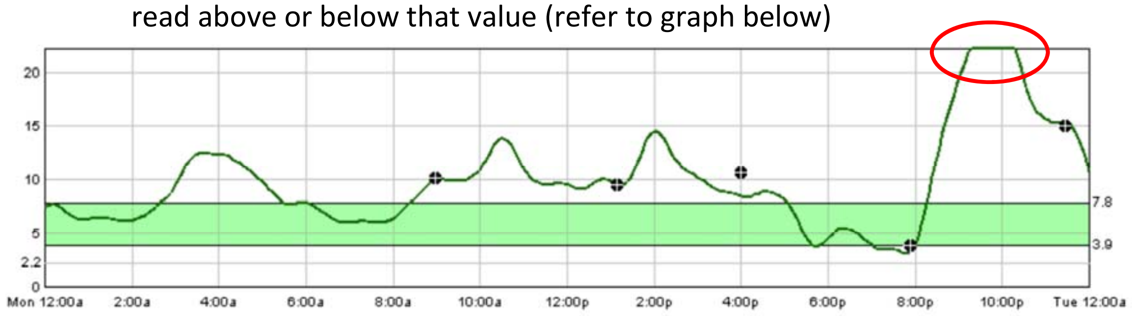

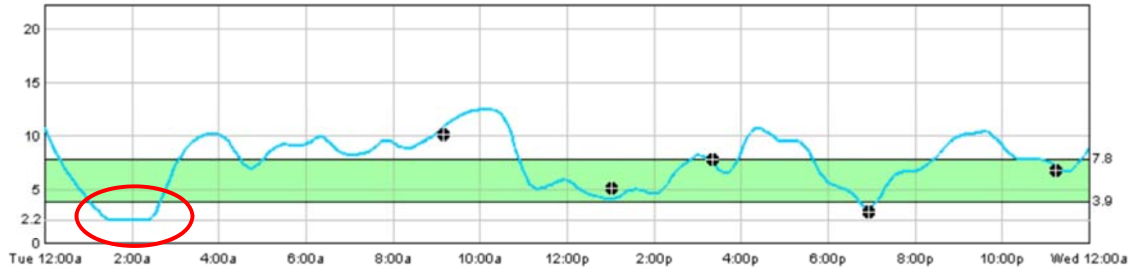

Guidelines for Insulin Adjustments and Glucose Supplementation

Insulin and Glucose Guidelines for Exercise

The study doctors have put together a list of recommendations for participants to follow regarding insulin adjustments and carbohydrate supplementation before, during, and after exercise.

Pre-Exercise Insulin Recommendations

- Avoid the following 12 hours before exercise:
  - Insulin injections in the legs.
- Avoid the following 2 hours before exercise:
  - Alcohol
  - Caffeine
  - Insulin (for example, if carbohydrates are consumed within 2 hours of exercise, avoid a correction dose)
- **MDI Patient:** decrease long-acting insulin dose by 10% (either the night before or the morning of the exercise session) and adjust accordingly with fast acting insulin correction doses throughout the day to avoid excessive hyperglycemia
- **Insulin Pump Patient:** decrease basal rate by 50% two hours before exercise, and maintain reduced basal rates until the end of exercise

Exercise/Testing Session Recommendations

- Blood glucose should be between 5.7-11.0 mmol/L prior to starting each exercise session
  - If blood glucose level is **lower than 5.7 mmol/l** prior to starting exercise, glucose should be consumed
  - If blood glucose level is **higher than 15.0 mmol/l** prior to starting exercise, test for ketones and wait for 30 minutes to see if values fall below 11.0 mmol/L, if it does not, reschedule the exercise session.
  - If blood glucose level is **higher than 20.0 mmol/l** prior to starting exercise the session will be re-scheduled

During Exercise

- If blood glucose drops to 3.9 mmol/L or less, participant must consume carbohydrates (they can keep exercising at this point). Ask participant how much they would consume.
- If blood glucose drops to 2.9 mmol/L OR 3.9 mmol/L and they have symptoms of hypoglycemia, STOP exercise and consume carbohydrates. If blood glucose reaches >3.9 mmol/L, exercise can start again.

Post Exercise

- It is recommended that participants reduce insulin dose prior to bedtime in order to prevent nighttime lows (lower basal rates by 20% between midnight and 3 am for a pump user, or decrease long acting insulin injection for an MDI user if they are taking it at night with boluses of fast acting insulin to correct when necessary).
- To the best of their ability, participants should wake up once at night to test blood glucose.

General Recommendations

- After experiencing the exercise protocols involved in the study, if subjects wish to make changes to their insulin doses, recommend they follow guidelines given by their diabetes care givers.

Data Entry

- Nothing developed yet – separate document???

Exercise Calibration, Testing, and Cleaning

Calibration

- Wear gloves whenever using equipment in the exercise lab, if not sure if gloves are contaminated just switch to new pair
- Turn on metabolic cart 30 minutes prior to test
- Ensure the heater light is on and the pneumotach is warm after 5 minutes
- While waiting assemble the mask (ensure the spiral diaphragms "spongy" part is fully on the white ring). Do not put the rubber mouthpiece on till after calibration (scuba piece)
- After 30 minutes perform Gas Calibration
    - **If testing all day re-run gas calibration after 4 hours**
    - Open True One Program on computer, open gas calibration window
    - Enter the barometric pressure (mmHg), temperature (Celcius) , Humidity (%)
    - Barometer gauge is located adjacent to the sink
    - Turn the tank ¼ turn to open and run the gas calibration and ensure the tank set to 3 PSI
    - If it is not, adjust pressure, cancel gas calibration, and re-run
    - Ensure the change in O2 gain, and CO 2 is less than 3% (the lower the better) Enter this in the calibration log
    - Click on the Save button and this will update the parameters
- Perform Flowmeter Calibration
    - Perform for every test as the flow characteristics of each mask are different
    - Place the 3L syringe on the desk, and attach the assemble Hans Rudolf valve
    - Open flow calibration window and ensure all the pressure, humidity and temperature settings are correct
    - Run the flowmeter calibration by clicking on Sample Baseline
        - The first 5 stokes are detection and flushes so you can do these strokes quickly.
        - For the final strokes you want to start slow and speed up.
        - Peak flow 1st stroke 50-80 L/min, 2nd >100, 3rd >150 L/min, 4th and 5th >200 L/min
    - Remember to fully extend the syringe as you are trying to check for leaks so each stroke should read close to 3.0L.
        - The difference between strokes should be less than 3% Enter this in the calibration log
    - Now you can place the final rubber mouthpiece on the mask and you are ready to perform a test.

Testing

Staff Member Roles

- One person standing on the right side of the treadmill adjusting speed and incline
- One person manning the computer – this individual will record all the information as indicated on the exercise protocol form, they will also ask the client how they feel on the RPE scale. Tell the participant how long they have left at the end of each stage. In the final 5 seconds of the stage, count down so the client knows the intensity will increase
- One person taking blood pressure

Procedure

- Ensure participant performs a blood glucose measurement approximately 30 minutes before test starts and record on CRF
- Check that the participant has followed pre-test instructions (no caffeine or large meal 2 hours prior, no alcohol or exercise 6 hours prior to test)
- Measure height and weight following the standard SOPs for these measurements listed earlier in this document OR if height and weight were taken earlier in the same visit, use that height and weight.
- Have subject change into comfortable clothes for exercising, and at the same time instruct them to put on a heart rate monitor.
- In the True One Window Select VO_2Metabolic Testing, Enter ID #, Age, Height, Weight, Test type, and staff initials
- **Explain the procedures of the test.**
  - Purpose of the test is to measure your body's oxygen consumption.
  - It will start off relatively easy and the speed and or incline will go up every three minutes until nine minutes. After nine minutes it will get harder every minute
  - Your goal is to last as long as possible; we will encourage you to work as hard as possible, but you decide when you are finished
  - The test may end for two reasons: (1) you decide to stop (2) we may end the test if you are no longer able to safely keep the correct pace, or if we see any abnormal changes in your heart rate or blood pressure
  - Things to expect: you will feel sweaty and warm, your breathing will increase, and tomorrow your legs may feel fatigued.
  - Things that you should let us know about: pain in the chest, jaw, or legs, feelings of dizziness, or feeling like you are going to be sick, let us know and we will stop the test (sign for this is hand across throat, or simply grab onto arm bars of treadmill)
  - During the test you will not be able to talk to us, and if you try to talk this affects the outcome of the test. Therefore we will use hand signals, thumbs up to yes, and thumbs down for no, hand side to side for maybe or "so-so".
  - We will also be asking how hard you feel you are working on a scale of 6 -20. 6 represents no exertion at all, 20 maximal effort. It is important to go with how you feel, and not how you think you should feel.

- **Do you have any questions?**
- Take pre-test heart rate (ensure it is <100 bpm) if not have them sit wait 5 minutes and re-take.
- Take pre-test blood pressure standing to demonstrate the position it will be taken during testing. (Ensure it is less than 140/90... either systolic or diastolic) If either is elevated, wait 5 minutes and re-take.
- Take blood glucose and record on CRF.
    - Rule: blood glucose must be 5.7mmol/l or higher to start the test
    - Rule: test should be re-scheduled if blood glucose is 20.0 or higher
- Allow the participant to walk on the treadmill without the equipment to get used to getting on, walking, and getting off of the treadmill. This will serve as their warm up – should last approximately 3 minutes at a self-selected speed.
- After the warm up, give the participant water, as their mouth might get dry.
- With participant standing on the treadmill, put on the headset, have the participant put in the mouthpiece (like a snorkel), and instruct them to keep a tight seal throughout the test. Hook the clear tube to the mask, and put on the nose plugs (ask them to try to get air out their nose – they should not be able to if it is on properly).
- In the metabolic testing screen where subject ID, height and weight were previously entered, press OK. Watch the O_2 and CO_2 values rise and then fall.
- When O_2 and CO_2 values stabilize, have the participant straddle the treadmill
- Instruct the participant they can begin walking once the belt starts moving
- Increase the speed of the treadmill to 2.0 mph
- Once the participant is walking at 2.0 mph start the exercise test by pressing “Start Test” on the computer
- Record their heart rate every minute – you can look at the heart rate on the computer. If the “H” (located on the *right* side of the screen) is flashing it indicates that the heart rate monitor is picking up the participants heart rate.
- If the “H” stops flashing you may need to adjust the HR monitor on the participant, or swap the piece that clips onto the front of their heart rate monitor strap.
- Record their RPE and blood pressure as indicted on the exercise protocol form (RPE is recorded at the end of every stage, and blood pressure is recorded at minute 3, minute 6, and cool down).
- As the participant progresses through the stages, be sure that 2 staff members are prepared to catch the participant if necessary by placing their arm behind the participant.
- When the subject reaches volitional exhaustion, press “End Test”, “print text report”, and allow them to cool down at a self-selected speed.
- Be sure to take at least two blood pressure measurements during recovery to ensure blood response is normal
- Please refer to the chart on the next page to see when to record all the information mentioned above.
- During the test be sure to write down notes either on the back of the exercise recording sheet or on a piece of paper. Things to take note of would be:

- If the client had to stop for any reason (sore calves, blood sugar was too low/high)
- If the heart rate was incorrect on the computer
- Anything else deemed important to take note of

**ABSOLUTE INDICATIONS TO STOP TESTING**
Drop in systolic pressure of >10 mmHg from the baseline value with increasing load (taken in same position of the test) when accompanied by ischemic symptoms
Moderate angina (chest pain)
Ataxia (coordination issues), dizziness
Signs of poor perfusion (turning blue)
Technical difficulties with measuring BP
Participant wishes to stop
ECG changes: (ventricular tachycardia, ST elevation)

**RELATIVE INDICATIONS TO STOP TESTING**
Drop in systolic pressure of >10mmHg from baseline, with increasing load without evidence of ischemia
Fatigue, shortness of breath, wheezing, leg cramps
Increasing chest pain
Hypertensive response : SBP >250mmmHg, or DBP >115 mmHg

Cleaning

- Fill sink with warm soapy water, and place all the materials to soak for 15 minutes.
- Rinse in warm water and place in Cidex solution for 15 minutes. ** Ensure date is correct on Cidex (2 week window of use)**.
- After 15 minutes in the Cidex, place material in the water bin for minutes. After 15 minutes place materials on the drying rack.
- Wash the heart rate strap (without the WearLink), head piece, and air tube by hand with the warm soapy water, and then empty the sink. Rinse thoroughly and place on the drying rack.
- After drying wipe down all the pegs used, and the equipment (headset for the mask, treadmill/bike)
- Re assemble valve

Maximal Exercise Test for Baseline Visit

Time	Speed	Incline	RPE	HR	Manual BP	Comments
-15MIN						Pre BG readings:<br>Reading 1: __ __ . __ mmol/L
IMMEDIATELY BEFORE TEST START						Reading 2: __ __ . __ mmol/L
0-1:00	2.0	0				
1:00-2:00	2.0	0				
2:00-3:00	2.0	0				
3:00-4:00	3.5	0				
4:00-5:00	3.5	0				
5:00-6:00	3.5	0				
6:00-7:00	5.0	0				
7:00-8:00	5.0	0				
8:00-9:00	5.0	0				
9:00-10:00	5.0	2.0				
10:00-11:00	5.0	4.0				
11:00-12:00	5.0	6.0				
12:00-13:00	5.0	8.0				
13:00-14:00	5.0	10.0				
14:00-15:00	5.0	12.0				

Exercise Session

There will be a total of 4 exercise sessions:

1. Moderate intensity exercise 45-55% max fitness level
2. Moderate exercise + bouts of 70% max fitness level
3. Moderate exercise + bouts of 80% max fitness level
4. Moderate exercise + bouts of 90% max fitness level

(Sessions 2–4:) These sessions will be randomized

Procedure

1. Develop rapport with the participant upon their arrival and explain to them what to expect during the exercise session. Make sure they consumed their Glucerna bar either before or upon arrival at the laboratory. Make note of any adjustments they might have made to their insulin to account for it.
2. Upon arrival the participant should take one finger poke using a handheld glucometer
    a. Their blood glucose MUST be >5.7mmol/L but <20.0 mmol/L prior to starting exercise. At least 2 capillary glucose readings should be taken before exercise to ensure that glucose levels are in the correct range and are not showing a strong downward or upward trend.
    b. For more details refer to blood glucose and insulin recommendations as mentioned earlier in this document.
3. Measure heart rate, blood pressure, blood glucose and have the nurse take a blood draw immediately prior to the exercise session as indicated on the recording sheet on the previous page.
    a. The first blood draw will be taken while the participant is standing on the treadmill so they can get a sense of how to position their arm for further blood draws throughout the session.
4. Select the appropriate speed for the participant and instruct them to begin walking once the belt starts moving
    a. The intensity of each session will be based on the maximum value that was achieved during the fitness test.
5. The warm--up will last for 10 minutes and heart rate, blood pressure, blood glucose will be measured and a blood draw will be taken by a nurse.
    a. A summary of blood draw times include:
        i. Baseline (time 0)
        ii. End of warm up (time 10)
        iii. End of intervals (time 35)
        iv. End of exercise (time 45)
        v. Mid-recovery (time 75)
        vi. End-recovery (time 105)
    b. **Note:** additional blood draws may be taken if the participant's glucose levels are a concern. The sole purpose of these additional blood draws will be to verify venous glucose levels.
6. Each exercise session will last for 45 minutes (not including warm up or cool down).

a. The moderate intensity session will be 45 minutes of continuous exercise at 45-55% max fitness level
b. For the vigorous exercise sessions each bout of intense exercise at 70%, 80%, or 90% will last for 1 minute with 4 minutes of recovery between each bout. The first bout of vigorous exercise will occur after the warm up which lasts for 10 minutes.

7. Once the participant finishes the 45 minute exercise session, begin the recovery at a self-selected speed at 0% incline.

Exercise Session Recording Sheet: Moderate

Stage	Time	Treadmill speed	Treadmill incline	End stage heart rate	RPE	Blood draw time	Blood glucose*	Added Notes
Baseline	0					(0)		
Moderate Exercise 45-55% VO_2max	0-**10**					(10)		
	10-**15**						(15)	
	15-**20**							
	20-**25**							
	25-**30**						(30)	
	30-**35**					(35)		
	35-**40**							
	40-**45**					(45)		
						(75)	(75)	
						(105)	(105)	

Exercise Session Recording Sheet: Vigorous

Stage	Time	Treadmill speed	Treadmill incline	End stage heart rate	RPE	Blood draw time	Blood glucose*	Added Notes
Baseline	0					(0)		
Warm-up	0-10					(10)		
**Interval 1**	**10-11**							
Recovery	11-15							
**Interval 2**	**15-16**							
Recovery	16-20							
**Interval 3**	**20-21**						(21)	
Recovery	21-25							
**Interval 4**	**25-26**							
Recovery	26-30							
**Interval 5**	**30-31**						(31)	
Recovery	31-35**							
**Interval 6**	**35-36**					(35)		
Cool-down	36-45**					(45)		
Recovery 1	45-75**					(75)	(60, 75)	
Recovery 2	75-105**					(105)	(90, 105)	
				**Overall RPE**				

Important Things to Remember

- Be sure to ask the client how they are feeling throughout the test
- Because these sessions are longer than the fitness test, it is very important to monitor their blood glucose as some individuals are not as sensitive to the symptoms of hypoglycemia

Blood Sampling Timeline and Checklist

Prior to Participant Arrival

- Make sure that the research nurse is booked and confirmed
- Confirm freezer space in -80C freezer
- Label and set out all test tubes (participant ID, time of blood sample, session)
    - V____ - 1, 2, 3, 4, 5, *OR* 6 – M, 70, 80, *OR* 90
- Label and set out all microcentrifuge tubes (participant ID, time of blood sample, session, P (plasma) or S (serum))
    - V____ - 1, 2, 3, 4, 5, *OR* 6 – M, 70, 80, *OR* 90 – P *OR* S
- Depending on the centrifuge available, be sure it is on and set to the appropriate temperature/speed/time settings
    - Sorval ST 16R Centrifuge
        - 4 degrees, 10 minutes, 1500xg

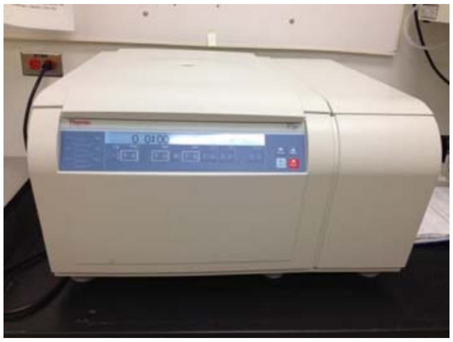

    - StatSpin Centrifuge
        - 3 minutes

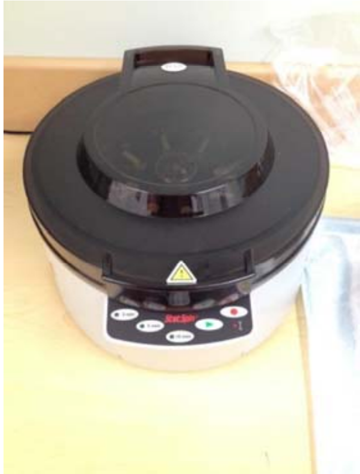

Supplies for Nurses

Ensure the following supplies are available in the testing room (use cart or trolley with bench pad)

- 20 gauge catheters (2)
- Catheter extension
- Leur-Lock Access Device (6)
- 10 cc syringes (12) – empty
- 5 cc syringes (12) – filled with saline
- Saline solution (2)
- Blunt fill needles (2)
- Gloves
- Plastic canulae (24)
- Tourniquet (2)
- Transpore Tape
- Sterile blue pad
- IV 3000 (2)
- Gauze pads
- Vacutainer tubes (12 red top tubes & 6 purple top tubes)
- Labelled micro tubes (12)
- Pipette
- Sharps container
- Alcohol swabs
- Band aids

For Samples Drawn at 0, 10, 35, 45, 75, and 105 minutes

- Research nurse will draw blood using vacutainers and hand to researcher(s)/assistant(s)
- Transfer blood to red and purple top tubes (purple = plasma, red = serum)
    - For each blood draw, 3 tubes will be filled (1 purple and 2 red) – the very first blood draw of the moderate session will use 4 tubes (2 purple and 2 red)
        - One set of red tubes will be sent to the lab in HSC and the other set of red tubes will clot for 20-30 minutes before they are centrifuged
        - The very first blood draw with the purple tubes – one tube will be sent to the lab in HSC and the other will be centrifuged
- Make sure the purple top tube is flipped 10 times before it is centrifuged
    - **The red top tube gives a better sample when it is spun for 12 minutes (Sorval ST 16R Centrifuge ONLY)**
- After tubes have been centrifuged pipette the clear supernatant and transfer to labelled micro tubes. Once the clear supernatant has been pipetted you can discard the red/purple top tubes into the biohazard bin.
- Discard all other blood-soiled instruments in the biohazard waste bins
- Immediately place micro tubes in the -20°C freezer OR a bucket of ice
    - Label the micro tube boxes as follows:

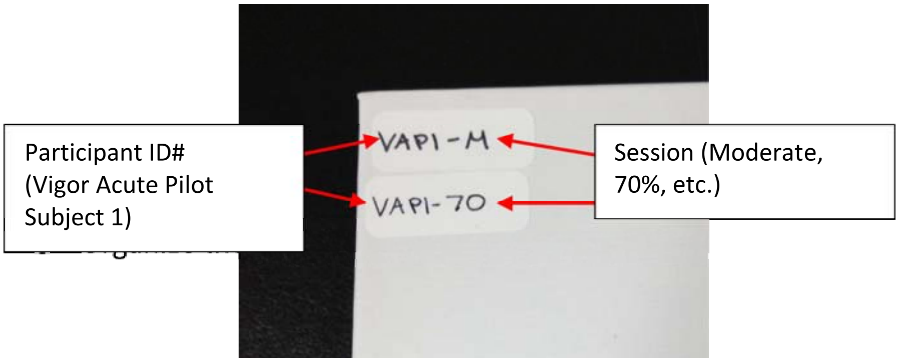

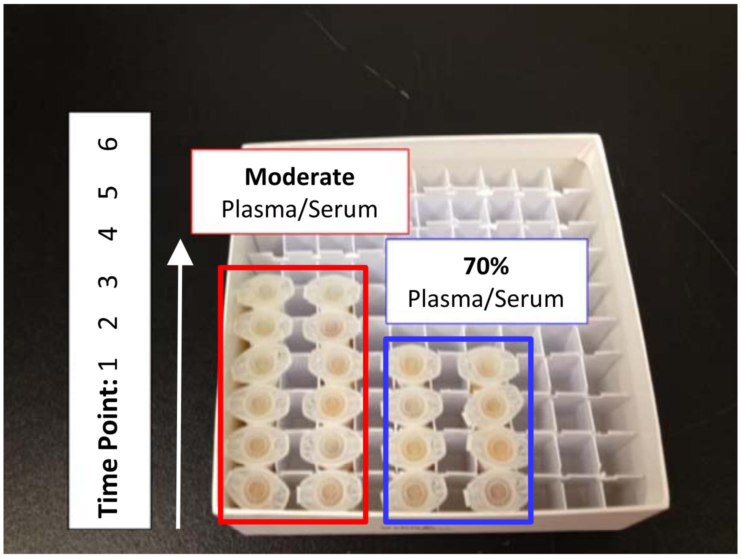

- The following day, transfer samples from -20°C freezer to -80°C freezer
- *Please refer to the timeline chart below*

Once the catheter is removed, have the participant apply firm pressure with sterile gauze for at least 5 minutes before applying a bandage to the wound in order to decrease the amount of bruising.

Special Note for Blood Draws

- If a catheter cannot be inserted the study nurse will use butterfly needles instead. This will require 4 blood draws with 4 different needles. The time points for blood draws will be:
    - 1 (Baseline)
    - 4 (End of exercise)
    - 5 (Mid-recovery) – if feasible
    - 6 (End-recovery)
- In addition to the blood draws, the participant will also need to take blood glucose with a handheld glucometer at:
    - 0 (Baseline)
    - 10 (End or warm up)
    - 15
    - 30
    - 60
    - 75 (Mid-recovery)
    - 90
    - 105 (End-recovery)

Time	Purple Top Tube	Done?	Red Top Tube	Done?
0	**Draw #1 -** put tube in centrifuge		**Draw #1 - let stand 20 minutes**	
5				
10	**Draw #2 -** put tube in centrifuge		**Draw #2 - let stand 20 minutes**	
15			Bring tubes #1 and #2 to lab	
20	Pipette sample #1			
25	Pipette sample #2			
30			Put samples #1 and #2 in centrifuge	
35	**Draw #3** -put tube in centrifuge		**Draw #3 - let stand 20 minutes**	
40			Pipette samples #1 and #2	
45	**Draw #4** - put tube in centrifuge		**Draw #4 - let stand 20 minutes**	
50			Bring tubes #3 and #4 to the lab	
55	Pipette sample #3			
60	Pipette sample #4			
65			Put samples #3 and #4 in centrifuge	
70				
75	**Draw #5** - put tube in centrifuge		**Draw #5**	
80			Pipette samples #3 and #4	
85	Pipette sample #5			
90				
95			Put sample #5 in centrifuge	
100				
105	**Draw #6** - put tube in centrifuge		**Draw #6**	
110			Bring tubes #5 and #6 to the lab	
115	Pipette #6		Pipette #5	
120				
125			Put sample #6 in centrifuge	
130				
135			Pipette #6	
